# The human cell count and size distribution

**DOI:** 10.1073/pnas.2303077120

**Published:** 2023-09-18

**Authors:** Ian A. Hatton, Eric D. Galbraith, Nono S. C. Merleau, Teemu P. Miettinen, Benjamin McDonald Smith, Jeffery A. Shander

**Affiliations:** ^a^Max Planck Institute for Mathematics in the Sciences, Leipzig 04103, Germany; ^b^Department of Earth and Planetary Sciences, McGill University, Montreal, Quebec H3A 0E8, Canada; ^c^ICREA, Barcelona 08010, Spain; ^d^Center for Scalable Data Analytics and Artificial Intelligence, University of Leipzig, D-04105 Leipzig, Germany; ^e^Koch Institute for Integrative Cancer Research, Massachusetts Institute of Technology, Cambridge, MA 02139; ^f^Department of Medicine, McGill University Health Centre Research Institute, Montreal, Quebec H4A 3S5, Canada; ^g^Department of Medicine, Columbia University Medical Center, New York, NY 10032; ^h^Independent Researcher and Consultant, Solon, IA 52333

**Keywords:** cell size, cell count, cell biomass, size distribution, size homeostasis

## Abstract

Cell size and cell count are adaptively regulated and intimately linked to growth and function. Yet, despite their widespread relevance, the relation between cell size and count has never been formally examined over the whole human body. Here, we compile a comprehensive dataset of cell size and count over all major cell types, with data drawn from >1,500 published sources. We consider the body of a representative male (70 kg), which allows further estimates of a female (60 kg) and 10-y-old child (32 kg). We build a hierarchical interface for the cellular organization of the body, giving easy access to data, methods, and sources (https://humancelltreemap.mis.mpg.de/). In total, we estimate total body counts of ≈36 trillion cells in the male, ≈28 trillion in the female, and ≈17 trillion in the child. These data reveal a surprising inverse relation between cell size and count, implying a trade-off between these variables, such that all cells within a given logarithmic size class contribute an equal fraction to the body’s total cellular biomass. We also find that the coefficient of variation is approximately independent of mean cell size, implying the existence of cell-size regulation across cell types. Our data serve to establish a holistic quantitative framework for the cells of the human body, and highlight large-scale patterns in cell biology.

## Significance

A consistent and comprehensive quantitative framework of the cells in the human body could benefit many areas of biology. We compile data to estimate cell mass, size range, and cell count for some 1,200 cell groups, from the smallest red blood cells to the largest muscle fibers, across 60 tissues in a representative male, female, and 10-y-old child. We find large-scale patterns revealing that both cellular biomass in any given logarithmic cell-size class and the coefficient of cell-size variation are both approximately independent of cell size. These patterns are suggestive of a whole-organism trade-off between cell size and count and imply the existence of cell-size homeostasis across cell types.

Cells can be considered the basic units of life. Each cell type typically has a characteristic size range (1–4), which is not only maintained in regenerating tissues, but also throughout individual ontogeny from birth to maturity, and is often highly constrained across mammals from mouse to elephant (5, 6) (*SI Appendix*, Fig. S1). Because of the uniformity of cell size for any given type, a larger body generally has a proportionately larger cell count (1, 5, 7), rather than being comprised of larger sized cells. It has also been shown that the sizes of cells within a given type are usually well described by a lognormal distribution, often ranging over about an order of magnitude (*SI Appendix*, Fig. S2). But despite the uniformity in size of any given cell type, cell sizes vary over an enormous seven orders of magnitude from red blood cells to the largest muscle fibers, comparable to the mass ratio of a shrew to a blue whale. This raises the question: What is the size distribution of cells over the entire human body? When viewed across all the cells of a body, should we expect a lognormal distribution, similar to that of a single cell type, or some other distribution that favors particular sizes and functions?

At one level, it is apparent that the body’s cells are the “right size” to best perform their function (2, 3, 7, 8). Irregularities in cell size are often a pathological sign of disease or a marker of malignancy (9, 10). Moreover, changes in cell size are associated with changes in biosynthetic capacity and metabolic function (8, 11–13), especially when cell-size changes are not accompanied by changes in ploidy (14, 15). Cell “types” such as myocytes, neurons, and adipocytes vary extensively in size, but each cell has a size specificity suited to its function (e.g., muscle and sensory cells are smaller in the face than in the legs). This size homeostasis is thought to be maintained to some degree across all cell types, but the strength of size regulation, which is often measured as the coefficient of variation (CV) in size, remains unknown across the full cell-size range.

At a higher level of organization, the overall functional capacity of a tissue depends on its total biomass. Since the cell sizes of any given type are relatively well constrained (*SI Appendix*, Fig. S2), a tissue’s functional capacity is determined principally by the total number of its cells (1, 5, 7). Two important studies in the past decade have surveyed the total number of cells over the largest tissues in the human body, with total counts converging on values of 30 to 37 trillion human cells (16, 17), with an approximately equal number of bacteria cells (17). Although cell size and number are direct corollaries of growth and proliferation (18, 19), and among the most fundamental quantitative attributes of life’s basic units, the relation between cell size and number has never been formally examined at the level of the whole body.

We compile cell size, abundance, and diversity across tissues to provide a holistic view of the body’s basic functional units (1, 2–4, 7, 8, 20). Such a whole-organism view is particularly timely given the recent plan to build a Human Cell Atlas (21–23). This initiative seeks to create molecular profiles of all human cell types, enabled by the rapid development of single-cell multiomics. However, there are challenges to integrate studies focused on tissue samples into a whole-body framework that is consistent with what is known from classical cell biology (22). By integrating existing histology and anatomy across broad cell types in all major tissues, we hope that our data might help to establish a quantitative foundation for all cells of the human body.

## Approach

We consider cell size and number for some 400 cell types in the bodies of three reference human anatomical models (24–26) (*Materials and Methods*). These include an adult male of 70 kg, an adult female of 60 kg, and a 10-y-old child of 32 kg. We base our analysis on the extensive catalogue of tissue masses and compositions compiled for the reference male model by the International Commission on Radiological Protection, ICRP (24–26). We then use the male model as the basis for estimating cell sizes and abundance values for the ICRP female and child models. We use tissue mass ratios reported by ICRP (24–26) to maintain correspondence with their models. In most cases, we assumed similar ratios among cell types, and similar cell sizes across the three models, the exceptions being neurons, adipocytes, and myocytes (*Materials and Methods*). We thus have greatest confidence in our size and count data for the male model, but uncertainty in the conversions from male to female and child are unlikely to be significant relative to other sources of error in the data.

Our data and methods build on prior work that estimated cell counts in approximately 20 tissue systems (16, 17). We partition the body into 60 well-studied tissue systems (24–26), each of which is further subdivided into major components (stroma, parenchyma, and extracellular matter) and dominant cell types. Such partitioning yields 1,264 separate cell groups, comprising the 400 major cell types across 60 tissues. For each cell group, we compile data from the literature to calculate cell-size mean and range, and to estimate their number across the three models.

Most of our estimates of cell mass are not direct mass measurements, but derive from microscopy or other inferences of cell dimensions that are then converted to mass based on specific densities for particular cell classes (e.g., 1.06 g/mL). While this approach is less accurate than direct mass measurements which can be taken on certain cell types in solution, these inaccuracies are likely to be minor relative to the trends that we observe over several orders of magnitude variation in cell size. Our classification of some 400 cell types is based on cell morphology and function (*Materials and Methods*) rather than molecular markers and RNA expression, which are currently resolving classical cell taxonomy into many additional cell types (27, 28). We use a combination of reported count or concentration, total aggregate mass, surface area, and/or DNA concentration to estimate the cell count for each cell type in each tissue. Our approach thus provides a consistent methodology applied to all cell types, including those less amenable to more modern techniques. We cross-validate our results by comparing multiple levels of aggregate mass at tissue and organ levels (24–26) (*Materials and Methods*).

## Results

### A Comprehensive Human Cell Dataset.

Our compilation of data presents an attempt to constrain cell parameters across all major cell types of the human body. A hierarchical interface to these data, giving methods and sources for each cell type in each tissue, is available at: https://humancelltreemap.mis.mpg.de/.

For several tissue systems, our compilation provides much greater resolution than was previously available, including muscle fiber sizes of all major striated muscle groups (*n* = 243 cell groups), neurons and glial cell groups throughout the central and peripheral nervous system (*n* = 244 cell groups), and the tissue resident blood cell groups (*n* = 172) from their origin in bone marrow, to their circulation and sequestration in the major blood organs and other tissues. We also focus on tissues of interest for human health, including digestive (*n* = 148 cell groups) and female reproductive tissues (*n* = 156 cell groups). While gaps remain (*SI Appendix*), these data present a detailed taxonomy of cell types across all major tissue groups and provide baseline values of cell size and count throughout the body.

**Fig. 1.**
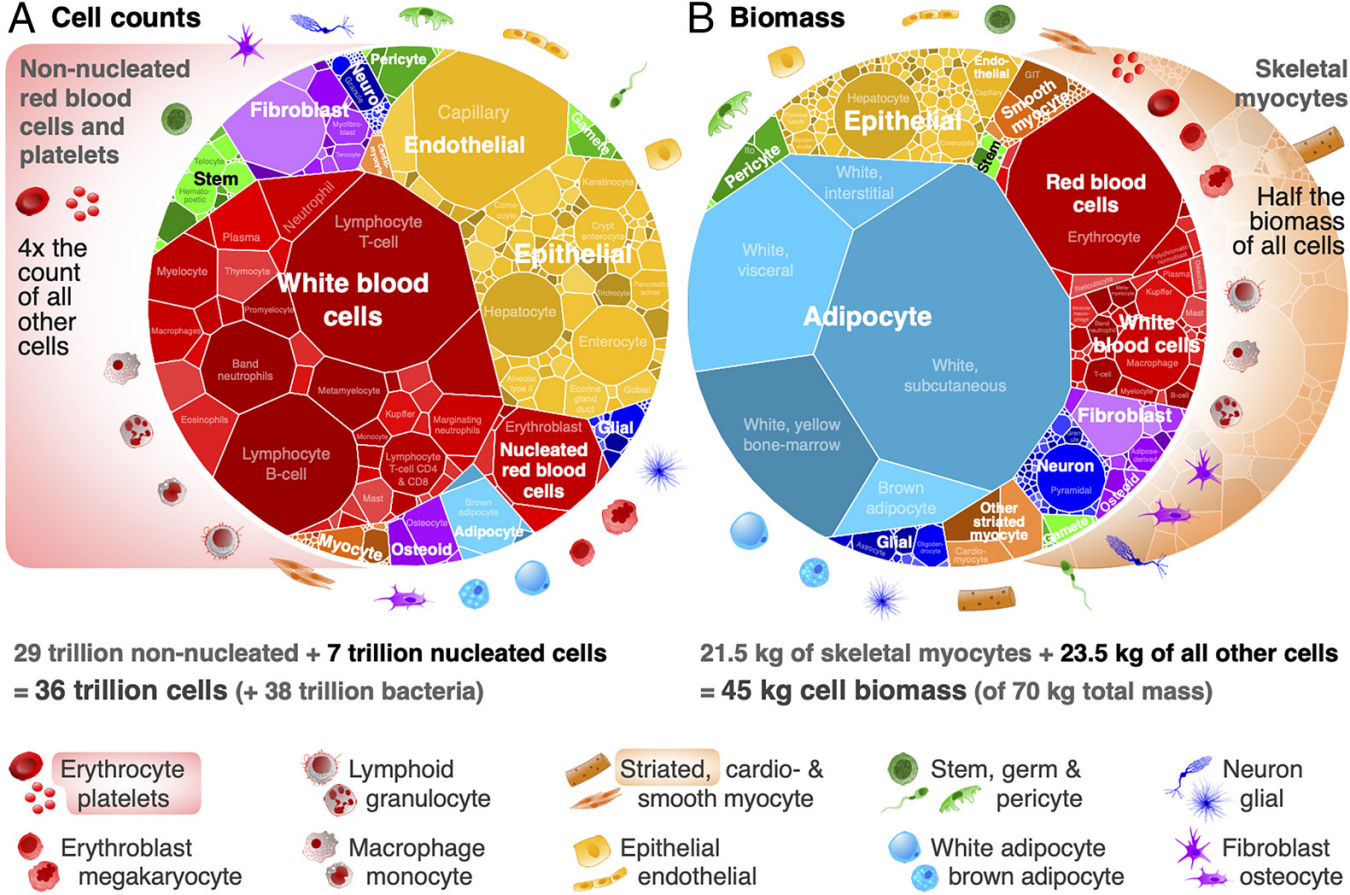
Contrasting cell count and biomass distributions by cell type. Voronoi tree maps for all 400 investigated cell types of the reference male anatomical model (area represents relative cell number or biomass). (*A*) Cell counts are dominated by red blood cells and platelets, which are removed from the cell count tree map. Even after removing nonnucleated blood cells (≈29 trillion), white blood cells (≈3.4 trillion) still dominate the ≈7 trillion nucleated cell count, with 98% of white blood cells as tissue resident, 1% circulating and 1% intravascular marginating. (*B*) Cell biomass is dominated by skeletal myocytes, comprising about half of all 45 kg of cell biomass in the body, even though they make up <0.002% of the nucleated cell count, which are removed from the biomass tree map. Most of the remaining 23.5 kg of cell biomass are white adipocytes (≈12 kg; though body fat varies widely among subjects). Tree maps for a reference 60-kg female (≈28 trillion cells) and 32-kg child (≈17 trillion cells), and more detailed cell groups and organ systems can be explored at https://humancelltreemap.mis.mpg.de/ (*SI Appendix*, Figs. S7–S9).

**Fig. 2.**
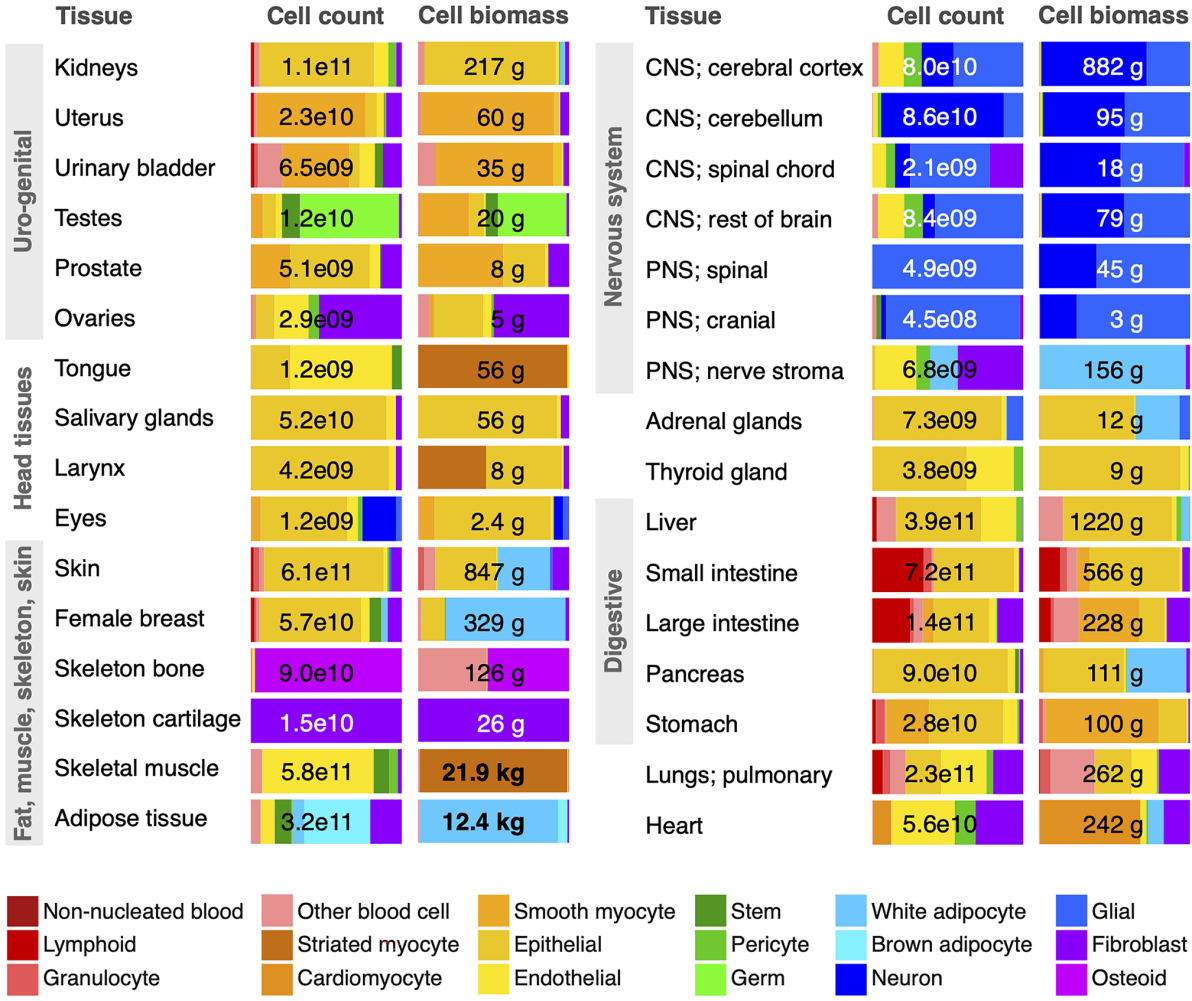
Cell class distributions across select tissues. Cell count and biomass distributions across 18 broad cell classes (colored) are shown for the 32 most significant tissue systems of the body, representing about half of all 60 investigated tissue systems, including the vast majority of total cell biomass. Numerical values refer to a reference male except for the female breasts, uterus, and ovaries. Most tissue systems are dominated by the ≈140 distinct cell types making up the epithelial cell class. “CNS” and “PNS” refer to central and peripheral nervous systems, respectively. The “Other blood cell” class is dominated by macrophages, but includes monocytes and precursors to red blood cells and platelets. Cell biomass excludes noncellular components of biomass in each tissue, made up of extracellular water, protein, and minerals (~25 kg; see *SI Appendix*). The blood cells of the blood organs and intravascular are shown separately in Fig. 1.

These data emphasize the vast size range of human cells, from a tiny red blood cell to a myocyte, more than a million-fold larger. Because of these vast size differences across some 400 major cell types (27, 28), we find dramatically different distributions between cell count and cell biomass (Fig. 1). Cell counts are completely dominated by red blood cells, platelets, and tissue resident white blood cells, while cell biomass is dominated by muscle and fat (myocytes and adipocytes). Within particular tissues, on the other hand, the dominant cell sizes vary much less, and so both count and biomass distributions are often quite similar (Fig. 2).

### Cell Size Is Near Inverse to Count.

**Fig. 3.**
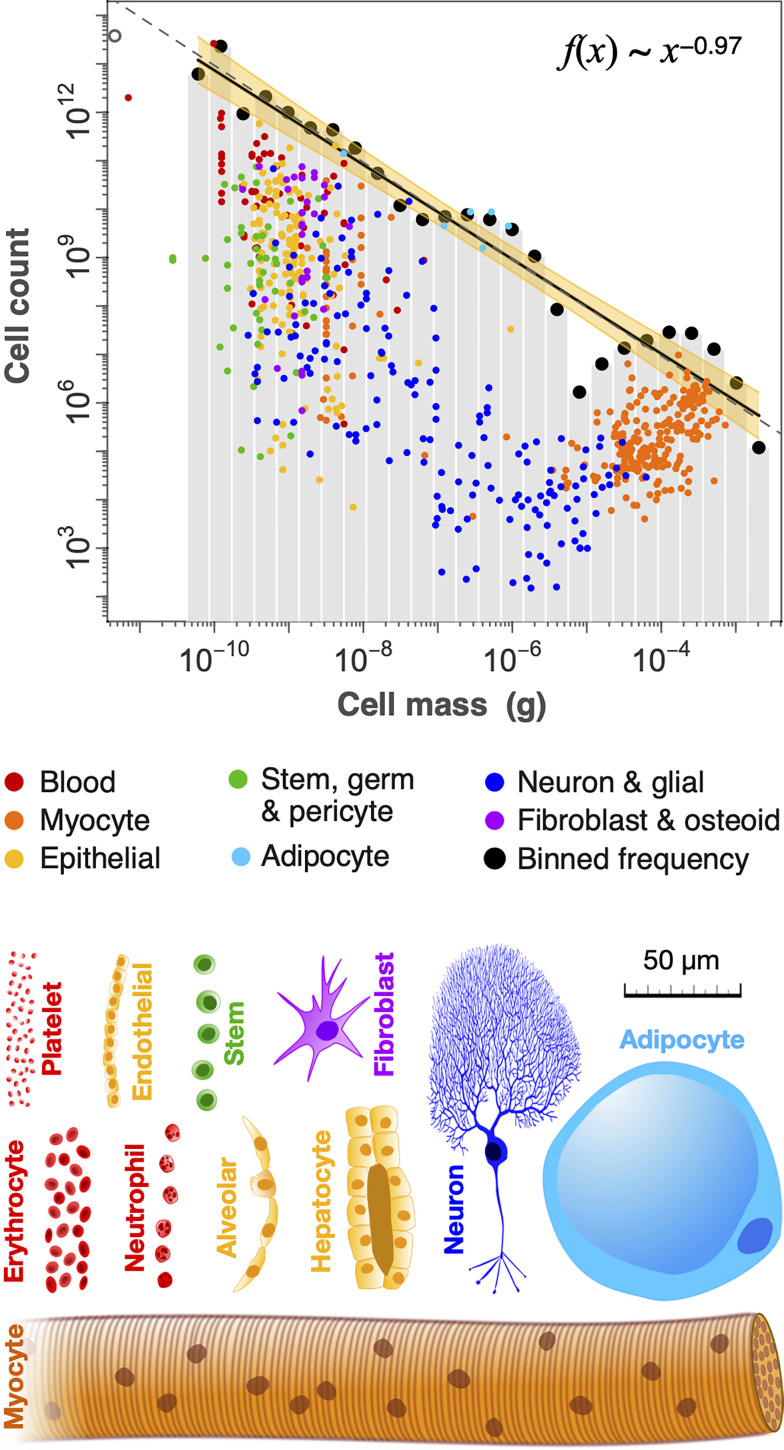
Human cell size and count are nearly inversely related. Data and regression are shown for a 70-kg reference male, though very similar patterns are found for female and child (*SI Appendix*, Fig. S5). The total number of cells in each of 26 logarithmic size classes is regressed against cell mass (g), giving a slope near −0.97, as shown by the black line fit to black circles. The yellow band shows the 95% CI (±0.1), while the dashed gray line shows a reference slope of −1. Small colored circles (*n* = 700) include 401 distinct cell types, in addition to single cell types that have large variation in size (e.g., adipocytes, neurons and myocytes). These points represent mean cell-size values for each cell group, and are aggregated over their size range into size class sums represented by black points (*Materials and Methods*). In some cases, cells with broad size distributions (e.g., adipocytes; light blue) have counts of mean sizes that are higher than the size class sum (black points), since the counts of actual sizes are spread lognormally over multiple size classes. The open gray circle (*Top Left*) is the bacterial microbiome (17).

A holistic view of cell size and count can be gained by constructing a cell-size histogram over the whole body. This distribution can be expressed in several ways, all of which yield similar results (*Materials and Methods*; *SI Appendix*, Fig. S4). The size distribution shown in Fig. 3 is built by spreading mean cell size of each cell type (shown by colored points in Fig. 3) over its size range, which sometimes spans multiple size classes (gray bars in Fig. 3). Given that a lognormal distribution provides a reasonable fit to prior published size distributions for most cell types (*SI Appendix*, Fig. S2), we assume a lognormal better describe each of the 1,264 cell groups in our dataset. We therefore partition cell counts lognormally to different size classes based on their reported range, and sum all cell counts over all cell types in each size class (*Materials and Methods*; *SI Appendix*, Fig. S3).

**Fig. 4.**
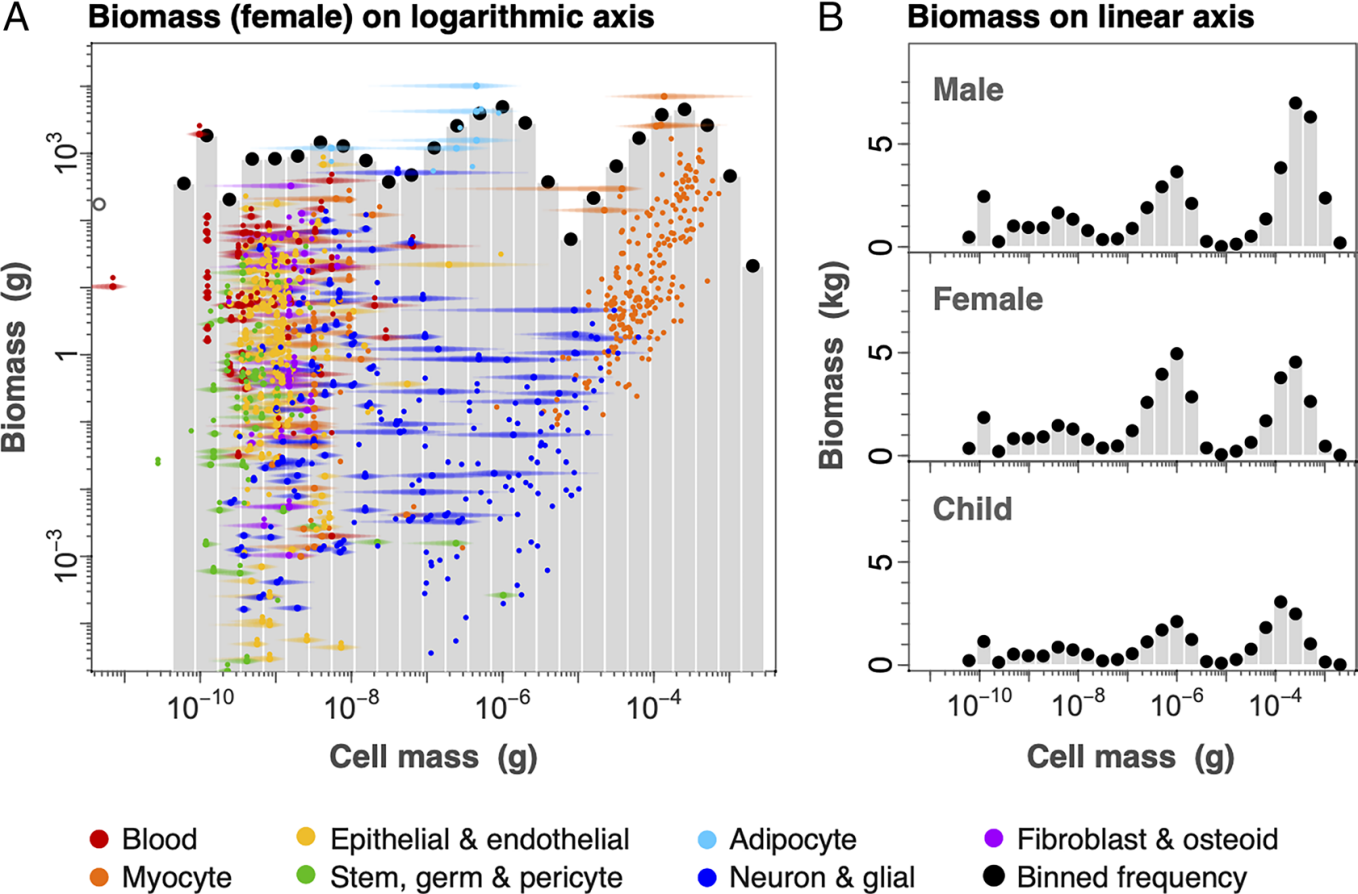
Cellular biomass and cell-size variation. The number of cells in a group multiplied by cell mass gives total biomass of a cell group. (*A*) Numerical counts (from Fig. 3) are transformed to total cellular biomass within a size class, and show little systematic variation with log cell mass. Here we show female cell biomass, which is broadly similar to the male and child (as shown in *B* on linear axes). Horizontal colored bands represent approximate size ranges based on literature values. In some cases, the biomass of a cell type (colored points) may be greater than the size class sum if that cell type is broadly distributed (e.g., adipocyte and myocyte). (*B*) Cell biomass can also be displayed on linear axes to highlight irregularities, particularly among the largest cell types (adipocytes and myocytes). Male, female, and 10-y-old child are broadly similar, though the child displays a slightly more even distribution of biomass (*SI Appendix*, Fig. S5).

We find a consistent relation between log total count in each log size class extending from red blood cells to the largest myocytes (Fig. 3). The slope of the relation is robust to alternative fitting methods and various binning schemes, and similar to the exponent of the corresponding cumulative distribution function, which avoids binning data into size classes (*Materials and Methods*). All approaches give exponents near the value of −0.97 and within the 95% CI reported in Fig. 3 (−1.09 to −0.87; *SI Appendix*, Fig. S4; *Materials and Methods*). A slope near −1 implies a roughly inverse relation between cell size and count, such that an increase in size by a given factor is offset by a decrease in cell count by a similar factor (and vice versa). Such an inverse relation implies that a roughly similar fraction of total body mass is present in each logarithmic cell-size class (Fig. 4*A*).

Although the cellular biomass is overall approximately evenly spread among cell-size classes (Fig. 4*A*), there are notable irregularities, which are more evident when plotted on a linear biomass axis (Fig. 4*B*). Among the smallest cells, there are breaks in the continuity of the size distribution around platelets and haploid sperm cells, near 10^−11^ g. Our size distribution does not include these small cells, and only considers the distribution beginning at red blood cells (4 × 10^−11^ g). There are also prominent biomass peaks comprising adipocytes (near 10^−6^ g) and myocytes (near 10^−4^ g), separated by a gap of relatively low biomass. Unlike most other cell types, adipocytes and myocytes have uncommonly broad size distributions and vary much more in size than in count, with cell counts remaining relatively fixed through ontogeny, obesity, or muscle atrophy (29–31). This gap is also evident but less prominent in the reference female, and is even less evident in the 10-y-old reference child (Fig. 4*B* and *SI Appendix*, Fig. S5). We expect that this gap may be even smaller in earlier ontogeny, when the musculature is less developed. With the exception of this gap, all variation in cellular biomass lies within two orders of magnitude while cell size varies over more than seven orders of magnitude. Despite the irregularities, these results suggest little systematic tendency for cellular biomass to vary across size classes.

### Cell-Size Variation Across Cell Types.

**Fig. 5.**
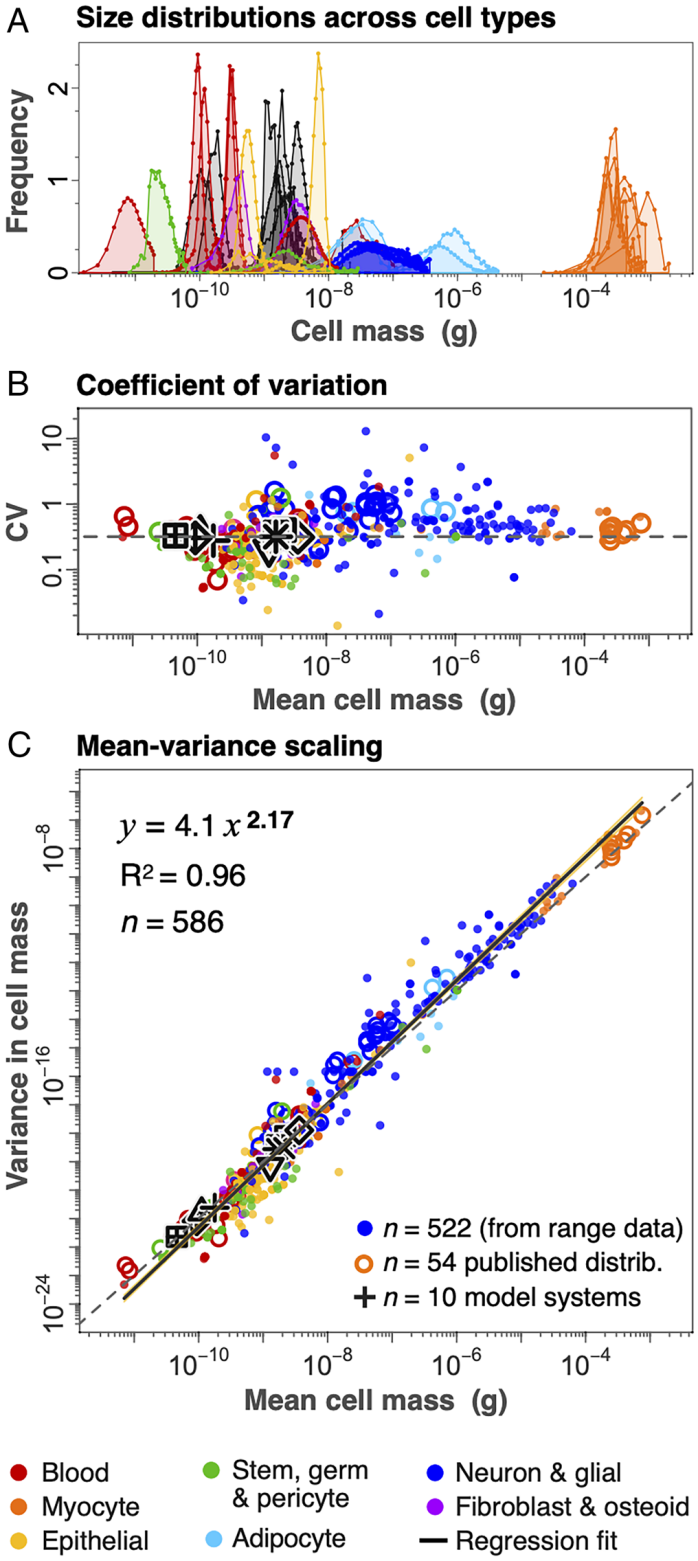
Size variation across cell types. (*A*) Published size distributions of select human cell types (colored; *n* = 30) and cell-size model systems (gray; *n* = 10) are normalized and found to be well approximated by a log-normal distribution (*Materials and Methods*; *SI Appendix*, Fig. S2). (*B*) The CV (SD divided by mean cell mass) shows little tendency to vary systematically with mean size across the full range of cell types. (*C*) The variance in cell mass for each cell type scales as a power law with exponent 2.17 (±0.04) against mean cell mass across all cell groups. Small solid points in *B* and *C* derive from estimates of SD from size ranges in our dataset (*n* = 522). Open circles (*n* = 54) are from prior published distributions and black shapes (*n* = 10) are from cell-size model systems shown in *A* (see *SI Appendix*, Fig. S2 for legend, and *Materials and Methods* and *SI Appendix* for sources).

Our data also allow us to consider variation in cell size across cell types. For cell groups with sufficient data, we use size range values across 522 different cell groups to estimate their CV (SD divided by mean cell mass; Fig. 5*A*). These estimates roughly align with the CV of prior published human cell-size distributions (*n* = 54; *SI Appendix*, Fig. S2), as well as ten classic model systems commonly used in cell-size research, and for which numerous high quality measurements exist (Fig. 5*B*; *Materials and Methods*). These distinct data sources all show that across the full suite of cell sizes, CV is approximately independent of mean cell size (Fig. 5*B* and *SI Appendix*, Fig. S6). This implies that cell mass variance should scale with the mean following a power law exponent of 2, and we estimate a value of 2.17 ± 0.03 (Fig. 5*C*; *Materials and Methods*). Despite adipocytes and some neuron cell groups displaying higher variability than most other cell types, there is not significant systematic variation in CV across cell types over the full size range. Any constancy in CV may imply that the underlying mechanisms responsible for cell-size homeostasis are present across cells in the human body (*SI Appendix*, Fig. S6).

## Discussion

### Data Uncertainty.

The data on which our study relies are often coarse and include several types of error. For example, many of the cell-size measurements rely on 2D microscopy and approximations of cell shape, rather than direct mass measurements, which are available for some cell types that can be isolated. For many cell types, there are limited available literature sources, and estimated mean sizes sometimes differ by as much as fourfold between sources. To express the uncertainty in cell size, we report upper and lower bounds on cell size (for 1,020 of 1,264 cell groups), which indicates the potential cell-size errors in the data (*Materials and Methods*).

Estimation errors are also evident in cell counts, which can vary as high as 20-fold between studies, though a factor of twofold to fourfold variation is more common. Examples of large variations in data between studies include the small intestine enterocytes and endothelial cells, as well as counts in the heart, adipose tissue, and lymph nodes (*Materials and Methods* and *SI Appendix*). It is not known how these errors might affect the noise observed in our analyses, be it irregularities in Fig. 3, or variability in Fig. 5. Nor can we be certain whether these differences reflect biological differences or measurement error. Nonetheless, we do not expect that the range of estimation error, typically less than a factor of 10, to significantly alter the broad-scale patterns we observe over seven orders of magnitude.

**Fig. 6.**
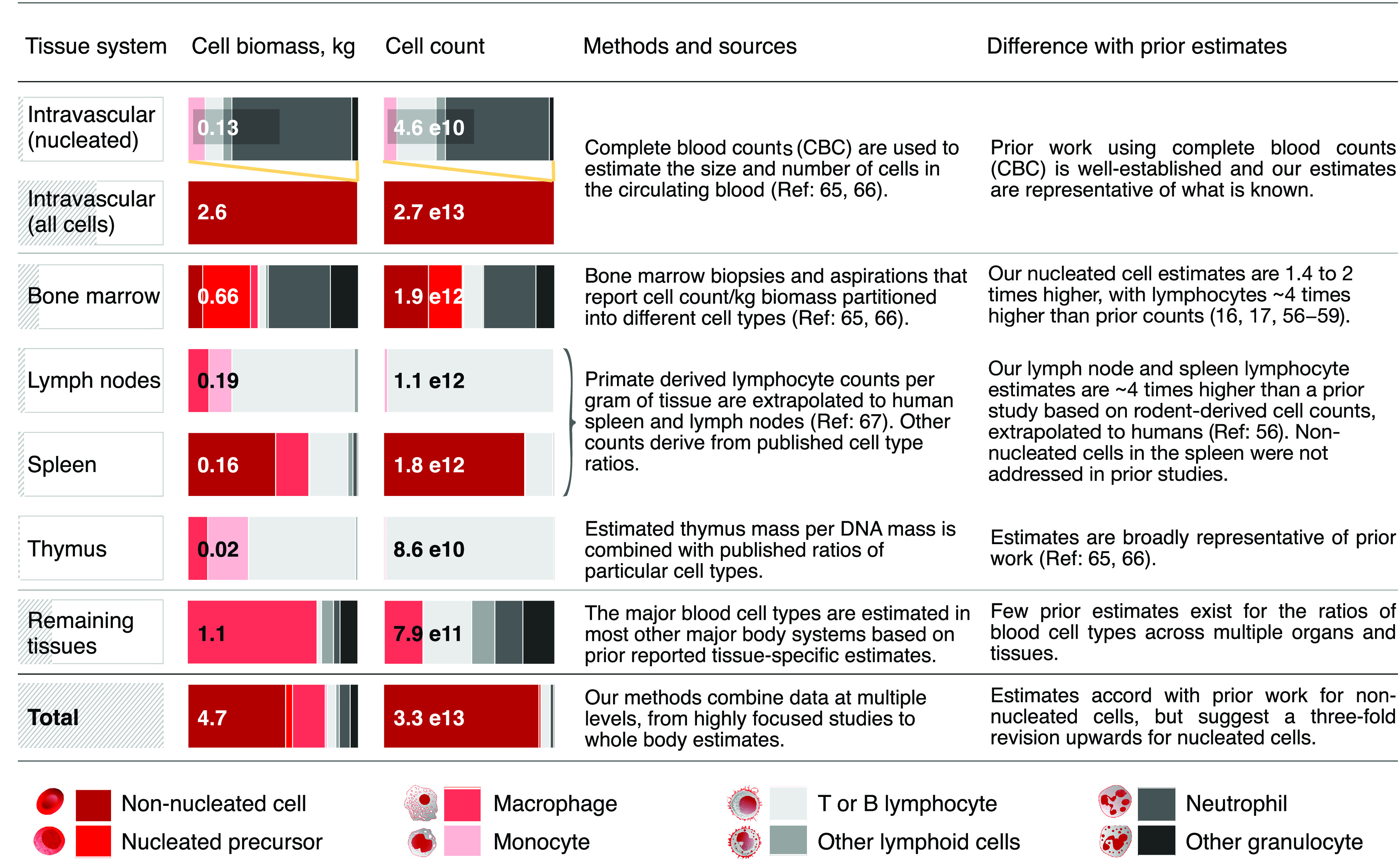
Summary of blood cell distribution across blood organs and other tissues. Blood cell size and count are estimated for each of the intravascular, major blood organs and over all remaining tissues. Numerical values refer to a reference male. These estimates derive from reported total biomass of each major tissue system. The relative biomass fraction for each tissue is shown as a hatched shaded gray bar in the leftmost column. The first row (intravascular) shows the same data as the second, except that nonnucleated red blood cells and platelets are removed (as in Fig. 1*A*). Total cell biomass (kg) and cell count in each system are partitioned into eight blood cell groups (colored bars) based on a variety of methods. We highlight where our methods and estimates differ with prior published sources.

Estimates for a number of cell types are prone to significant uncertainty. These include the size and counts of neuron and glial cells, and particularly the neurons of the peripheral nervous system. Stromal cells in general and vascular cells in particular also pose challenges to whole-body estimation, given their ubiquity throughout the body. Finally, our counts of the nucleated blood cells such as lymphocytes, are several fold higher than some prior estimates, a difference that should be further researched (Fig. 6). A broader discussion of major uncertainties is in *Materials and Methods* and *SI Appendix*.

### Cell DNA Content.

We have not investigated the relation between cell ploidy and cell mass, which has long been thought to vary proportionately (32), or possibly as a sublinear power law (20, 33). There are however, some significant exceptions to these relations. For example, some of the largest cells, such as adipocytes and neurons are typically diploid, while several smaller cells such as keratinocytes and pancreatic acinar and beta cells may be polyploid. It is also not yet clear how these relations might extend to the myonuclear domain.

We can, however, make a rough estimate of the proportion of polyploid cells throughout the body from ploidy estimates across major cell types. We estimate only about 1% of all nucleated cells are polyploid (see Dataset S1 for details). However, myocytes, each with thousands of diploid nuclei (31), make up about half of all cell biomass, such that polyploid cells dominate the cell biomass in the body. In total, polyploid cells account for about 10% of the body’s DNA.

### A Recurrent Pattern.

The near-inverse relationship between cell size and count that we report in Fig. 3 is reminiscent of classic experiments in particular tissues where cell size and number trade-off with one another to preserve tissue size. As mentioned, in some cell types, a doubling of cell ploidy can approximately double cell size, but the effect this often has is to halve the number of cells. This size-count trade-off maintains overall tissue biomass, as has been observed in salamanders (34), compartments of the *Drosophila* wing (35), mouse embryos (36), mouse liver (37), and possibly during human liver regeneration (38, 39). A plot of cell size vs. cell number across different experimental ploidies would thus reveal an inverse relation. The trade-off between cell size and count that maintains a relatively constant tissue size thus bears similarity to the inverse size-count pattern over the whole body (Fig. 3).

A similar pattern may hold across the bodies of mammal species. From mouse to elephant, the mean size of most cell types is similar (*SI Appendix*, Fig. S1) (5, 6), while most major tissue masses are roughly proportional with body mass (40). If we assume that the ratios of different cell types in different tissues are constant across species, we should expect a similar inverse cell size-count relation to hold in other mammals. As we have also suggested, there is reason to expect the same pattern may exist in infants. Taken together, these expectations suggest that predictable patterns between cell mass and count may be established across mammalian developmental programs.

This size distribution appears to be common in nature. The data in Fig. 3, can be expressed as a cumulative distribution function, which has an exponent α ≈ −0.95 (*SI Appendix*, Fig. S4*C*). A cumulative distribution function is proportional to a rank-size relation (*Materials and Methods*), and highlights a parallel to what is often termed Zipf’s law in other fields (41, 42). This pattern includes such disparate phenomena as the size distribution of cities (41), of asteroids (43) and of bodies in the ocean from bacteria to whales (44). Moreover, this general size-distribution has been previously associated with mean- variance scaling (45, 46), similar to what we show in Fig. 5*C* (*SI Appendix*, Fig. S6). Such power law scaling between the mean and variance in the size of a system is widely observed, particularly in the sizes of animal populations, and is known as Taylor’s law in ecology (47, 48). Both Zipf’s law (Fig. 3) and Taylor’s law (Fig. 5*C*) are common among populations of individuals (44, 47, 48), including unicellular systems (45, 49, 50). However, we are not aware of these patterns having been previously reported below the level of the individual, which highlights a recurrence across levels of organization.

### Origins.

To our knowledge, there is no universally recognized process that accounts for these patterns. Nonetheless, many simple random growth processes are capable of giving a stable inverse size distribution (41, 42, 51). In particular, because cell size and count emerge from the interplay of cell growth and division, the balance of these respective rates may prove to be an important factor. For balanced growth, rates of enlargement must match rates of division (52–55). Mismatches between these (possibly exponential) rates have the effect of multiplicatively varying size while, inversely, varying count. A relatively fast division rate results in both smaller cells, and more of them (and vice versa). For example, this distribution might emerge if we assume mismatches in these rates vary in such a way as to preserve mean cell size. If we further assume a lower (reflective) bound on cell size, then, following arguments in ref. 41, we should expect a size distribution with exponent −1. We emphasize that many simple mechanisms are possible, and no doubt the actual process is likely a great deal more complex. Nonetheless, the observed regularity between size and count may reflect fundamental principles that underlie the balanced control of cell growth and proliferation.

### Applications.

Our data may help to place overall bounds on human cell quantities and to identify major areas of uncertainty, as we summarize further in *SI Appendix*. For example, the total number of lymphocytes, often considered to be near 500 billion (56–59), is of key relevance for understanding immune function. Our assessment of the available data and more up-to-date studies of the blood organs and other tissues, suggests that this number may need a fourfold revision upward to approximately 2 trillion (*Materials and Methods*, *SI Appendix*). Such a major discrepancy of lymphocytes in the major blood organs could have implications for diagnostics of lymphocyte kinetics in leukemia, HIV, and other lymphocyte-related illnesses (60).

Our compilation of size ranges across cell types are rough, sometimes deriving from a small number of literature values. Nonetheless, they provide some constraint on expected cell size, which is often needed for cell identification in immunohistochemistry, cell isolation, or predictions on successful flow in microfluidics. More generally, an integrated framework of the prior literature facilitates contextualization of more focused studies, such as those pertaining to the Human Cell Atlas (21, 22). For example, our data could provide reference values in cases where different sequencing methods give different cell proportions over similar tissues (21, 23, 61). We also expect these data may help in understanding protein content and turnover, dosimetry, development of therapeutics, and as a baseline reference for cell biology (7). Finally, cell size may offer energetic and dynamical predictions, in the same way that body size predicts rates of growth, metabolism, maturation, and mortality at a much larger scale (62, 63).

### Conclusion.

We have attempted to summarize the basic quantities of size and count over most major cells of the human body. We show that cell biomass is roughly independent of cell-size class. Although cell size is often tightly regulated and closely linked to function (1–4, 9, 11–13), this independence suggests that there is no best size for a cell. Rather, the cells of the body are roughly spread across the cell-size range, through a trade-off between cell size and count. While the developmental mechanisms underlying this pattern remain unresolved, its similarity to patterns at other scales and in other fields is intriguing and may offer clues to its origin. Our holistic perspective of cell size and count has identified some major gaps in knowledge (*SI Appendix*), some of which may have health implications, such as the total body lymphocyte count. More generally, cell size and count are intimately linked to the dynamics of growth and proliferation (18, 19). A better understanding of the relation between cell size and count may thus yield insight into balanced growth as well as abnormalities in its regulation.

## Materials and Methods

Below we provide a high-level summary of the methods used to build our datasets and our approach for representing these data in the figures. More details are available in *SI Appendix*, which includes additional methods, major outstanding uncertainties, the differences with prior whole body cell counts, further notes to Figs. 1–6 and *SI Appendix*, Figs. S1–S9.

All data and detailed methods and original sources are available in Dataset S1, with supporting data in Dataset S2. An interface to Dataset S1 is at https://humancelltreemap.mis.mpg.de/.

To estimate the human cell-size distribution, we compiled measures of cell size and count across cell types and tissue groups of the bodies of three reference anatomical models (24–26):

– Male, (70 kg, 176 cm in height and 20 to 50 y of age).
– Female (60 kg, 163 cm in height and 20 to 50 y of age).
– Child (32 kg, 138 cm in height and 10 y of age) (24–26).

Our main dataset (Dataset S1) compiles cell size and count data from more than 1,500 published sources for 400 known cell types across 60 tissue systems in the body, giving 1,264 cell groups. These cell groups are classified according to two separate taxonomies; one based on various levels of cell type groupings, and one based on the body and tissue system in which different cell types are located. Recent work is further refining cell types into many additional classes (22), and we also provide a more resolved cell type classification into 924 more specific cell types. Although this may better align cell type taxonomy with more resolved cell types based on molecular markers or RNA expression, we cannot keep pace with the speed with which new cell types are being described. Included in Dataset S1 is a worksheet that lists the levels of ploidy in common cell types, from which we estimated the proportions of polyploid cells throughout the body.

Supporting data (Dataset S2) include a dataset on cell-size distributions for 54 cell types compiled from the literature, from which we selected the 30 cell types shown in Fig. 5 and the 25 cell types shown in *SI Appendix*, Fig. S2. This dataset also includes the 10 model systems commonly studied in cell-size research, shown as black shapes in Fig. 5 and *SI Appendix*, Fig. S2. Finally, we include a dataset of cell size for 33 cell types across mammal species spanning the size range from mouse to elephant shown in *SI Appendix*, Fig. S1.

### Data Compilation.

In order to estimate the cell size and number for the different cell groups, we used tissue parameters for the reference male, which comprises considerably more extensive and detailed data than exist for the female and child models (24–26). Tissue level data and parameters were drawn from the publications of the ICRP (24–26). The ICRP provides a common unifying base for which separate tissue masses and associated surface area, hydration level, protein content, extracellular mass, and fat mass have been estimated from multiple autopsies, and in vivo MRI and CT-scan data.

Estimates of cell size and count for a reference female and child are based on the more complete reference male model, following several simplifying assumptions. For most cell types, we assumed similar cell sizes and similar ratios of different cell types in each tissue. This allowed us to scale male cell counts to female and child models on the basis of reported ratios of respective tissue masses in each model for most of the ≈60 major tissue systems. For cells such as myocytes and peripheral nerves, on the other hand, we assumed that cell count is constant (29, 30), while cell size was assumed to vary as a system enlarges. In these cases, we scaled cell size (rather than cell count) accordingly between reference models. Finally, we undertook a detailed study of female reproductive organs, with particular emphasis on the cell groups of the breasts (*n* = 74), uterus (*n* = 34) and ovaries (*n* = 48).

For each tissue group detailed in the ICRP, we determined the relevant cell types, their relative proportions, and the mean and range (where possible) of cell mass. For commonly studied cell types, this was determined by taking an average and range of values from multiple published studies. For poorly studied cell types, cell sizes were estimated from closely related cell types, or calculations based on multiple cell components or contextual indicators. For example, in the absence of published measurements for peripheral neurons or myocytes we used average estimates of axon or myocyte diameter, based on cells in similar locations with similar functions. Estimates of cross-sectional area were then multiplied by an approximate length, appropriate for the cell’s relevant location in the body. In the case of neurons, we added cell body and dendrite mass. We determine the mean and range of cell mass for each cell type, and we calculate the number of cells based on their aggregate cellular mass, surface area and/or DNA concentration. Finally, cell masses and counts are cross-validated against multiple sources over a nested set of boundaries across the tissue to organ hierarchy.

Below, we briefly summarize each major cell class, providing a more detailed summary of each cell class in *SI Appendix*. We focus on the reference male model, unless otherwise stated. Note that the ordering by cell class does not reflect the manner by which the data were originally compiled. Rather, as indicated above, the body was segmented into separate tissue groups, with relative proportions of all the different cell types in each tissue estimated in each component. We then scaled those proportions to the relevant mass across each of the three focal anatomical models.

#### Blood cells.

The reference male contains 4.7 kg of blood cellular mass, 55% of which resides in the intravascular, while 45% is extravascular tissue-resident. Roughly half of the tissue resident cell mass resides in the four blood organs, distributed as shown in Fig. 6. In Fig. 6, we summarize these data into eight cell classes across six broad tissue categories. We have classified blood cells into *n* = 53 blood cell types, and enumerated many of these types across some 30 tissue systems. In total, we consider 191 blood cell groups, much of the data for which derive from refs. 64–67. Our estimates of nucleated blood cell counts are significantly higher than prior estimates (56–59), and deserve further study (Fig. 6).

#### Myocytes.

Myocytes include striated skeletal myocytes and cardiomyocytes, and smooth muscle cells. We compiled separate myocyte mean sizes and counts for 243 skeletal muscle groups in the body addressing 350 muscle pairs. We assumed that skeletal myocyte counts are fixed across reference models (30), and scaled myocyte size based on the ratios of the total muscle mass of female (0.625) or child (0.393) to male. Cardiomyocyte counts are based on published counts of myocytes for specific sections of the heart (68–70), extrapolated to the entire heart. Cardiomyocytes in the reference female and child require scaling both fiber size and count in both models based on heart mass ratios and patterns of cardiomyocyte maturation and hypertrophy (71, 72). Smooth muscle is distributed throughout the body (*n* = 27 cell types and *n* = 41 cell groups), and made use of specific studies, where available, or else generic smooth myocyte sizes.

#### Epithelial and endothelial.

Epithelial cells make up the majority of the well-known cell types in the body (*n* = 142 unique cell types and 228 cell groups), and as such are difficult to adequately summarize. For many tissues, estimates were made of cell count using a generic cell apical surface area and a total internal and external tissue surface area. Wherever possible, microvascular studies providing capillary densities (mm capillary per mm^3^ of tissue) in different locations were used in conjunction with endothelial apical surface area (600 to 1,350 µm^2^), to put bounds on endothelial cell counts for different parts of the body. Given their ubiquity throughout the body, there remain large uncertainties in endothelial cell sizes and counts.

#### Stem, germ, and pericyte.

Our data include *n* = 39 stem cell types distributed across most major body systems (73 stem cell groups). We refer to germ cells as all potentially reproductive cells in the germline, thus including haploid gamete cells. The reference male germ cells refer to the various diploid and haploid stages of sperm formation (*n* = 13 cell groups), while the reference female germ cells refer to the various groups of oocytes (*n* = 10 cell groups). Our data estimate pericyte counts in 38 tissues based primarily on location-specific pericytes to endothelial cell ratios, the latter of which we have estimated separately.

#### Adipocytes.

The reference male potassium-based total cellular mass of adipocytes is 13.3 kg (24). This is distributed as hypodermal (≈6.5 kg), yellow bone marrow (≈2.2 kg), visceral (≈3.5 kg), and the interstitial adipose tissue in different organs (≈840 g), with an additional 300 g of stromal cell mass, partitioned proportionately across the four groups. These values differ significantly in female and child models, totaling 18.1 kg and 6.9 kg of adipocyte cellular mass, respectively (25). We assumed that the relative proportions of different cell masses of adipocytes were constant across all anatomical models. We thus scaled the adipocyte counts according to the relative adipose tissue masses (25). There is some uncertainty in the size distribution of adipocytes over the whole body, as discussed in *SI Appendix*.

#### Neuron and glial.

Estimates of neuron and glial cells require several kinds of methods and pose significant challenges. In total, our data list mean cell masses and cell counts for 57 neuron cell types (159 groups) and 22 glial cell types (81 groups). These are distributed across various locations of the central and peripheral nervous systems, as well as other tissue systems, particularly the enteric nervous system. We estimate a total of 88 billion neurons and 87 billion nonneurons, consisting of ≈53 billion glial cells, ≈4 billion microglial macrophages, and 30 billion other stromal cells (vascular) in the brain of the reference male. This is consistent with a 1:1 neuron to nonneuron ratio and a total brain cell count of 170 ± 13 billion (73, 74). For the reference female and child, we assumed the masses of glial cells in both the CNS and PNS were constant across all models, and scaled their counts by various tissue mass ratios (25, 75–77). A similar approach was taken for the neuron cells in the CNS, but for those in the PNS, we assumed cell counts constant and scaled neuron sizes by tissue mass ratios.

#### Fibroblasts and osteoid.

We have used specific values for cell size and count in different tissue systems, where available. We estimate fibroblast cell sizes and counts in 56 tissue systems, which include chondrocytes, tenocytes, keratocytes, reticular cells, etc. Fibroblasts and osteoid cell masses and cell type ratios were assumed constant across reference male, female, and child models, while counts were scaled according to relative cellular mass ratios of the relevant tissues.

A more in-depth summary of each of these major cell classes is available as *SI Appendix*, *Supplementary Methods*, and specific details for each cell type are available in Dataset S1 and at https://humancelltreemap.mis.mpg.de/.

### Whole-Body Cell-Size Distribution.

We considered the cell-size distribution in multiple ways. First, we simply summed all counts of each cell type whose mean size fell within each log size class (*SI Appendix*, Fig. S4*D*). Using only the mean cell size for a cell type, however, can distort the overall distribution if that cell type actually varies over a broad range of size classes, resulting in irregularities that are possibly artifacts. An alternative is to use both the cell-size mean (colored points in Figs. 3 and 4*A*) and range (light horizontal lines in Fig. 4*A*). To do so, we assumed that the size distribution within any particular cell group can be described by a lognormal (Figs. 3 and 4 and *SI Appendix*, Fig. S2; see “*Size Variation across Cell Types*,” below) (7, 78). We further assumed that the size range represents four SDs, since in many cases our range values derive in this way from reported SDs in the literature.

To spread mean cell size across a more realistic size distribution for each cell type, we obtained lognormal quantiles for the counts, and used these to build the whole-body size distribution in Fig. 3 (outlined in *SI Appendix*, Fig. S3). We verified that partitioning our counts over lognormally distributed sizes in this way conserved both the total cell count and total biomass (to within a few grams). While this method of partitioning counts across size classes makes full use of the data available, it makes little difference to the exponent of the distribution (*SI Appendix*, Fig. S4).

We report ordinary least squares regression statistics for the fit between the log of total count in each size class vs. the log of the geometric mean of the size class. In the case plotted in Fig. 3, bins increase by a multiplicative factor of 2 from small to large (octave size classes), beginning at the lower bound of erythrocytes, and giving 26 size classes across the full range of cell sizes. We ignored binning cells smaller than erythrocytes (blood platelets and the smallest haploid gamete cells), to avoid fitting the distribution over relatively large gaps. In Fig. 3, we report that the distribution is well approximated by a power law with a slope near −0.97 with a 95% CI of −1.09 to −0.87.

Several alternative representations and fitting methods of the cell-size distribution give exponent values that are typically within our reported CIs (*SI Appendix*, Fig. S4). Varying size class width or bin number, and the way in which we distribute counts for each cell type over their size range, all yield exponents typically near −1 (*SI Appendix*, Fig. S4 *B* and *E*). As an alternative to binning the data into size classes, we can represent cell sizes as a complementary cumulative distribution function (CCDF) (42). Plots of the CCDF are shown in *SI Appendix*, Fig. S4 *C* and *F*. While we have reported CCDF exponent values derived from least squares, we have also fit these distributions using maximum likelihood, using methods for binned data (79), finding that the fitting method does not significantly alter the exponent, with best fits within our reported 95% CI.

The cumulative distribution function (CCDF) is the fraction (or else the total number) of cells with size greater than or equal to *x*. The CCDF, with exponent α ≈ −1, means the probability that the size of a cell is greater than some *x* is proportional to 1/*x*; that is, *P*(size > *x*) ~ *x*^α^. The cumulative distribution is proportional to another common representation, which is the rank of cell size. A rank-size relation is the way that Zipf’s law is most typically represented (41, 42). If we rank the sizes of cells from smallest to largest, then there are *n* cells with size greater than or equal to that of the *n*th largest cell. But “the *n*th largest cell is of size *x*” (rank), is the same as “*n* cells are of size *x* or greater” (cumulative distribution). As such, the exponent obtained from regressing the log count against the log size class (e.g. α ≈ −0.97, Fig. 3), should give a similar exponent to the CCDF (e.g. α ≈ −0.95; *SI Appendix*, Fig. S4*C*), or the rank-size relation. It should also be noted that the corresponding probability density has an exponent α −1; that is, *p*(*x*) ~ *x*^α−1^, thus having a value near −2.

### Size Variation Across Cell Types.

We obtained cell-size range data for 1,020 of 1,264 cell groups in our dataset, though many of these are the same for a given cell type in different tissues. These data were obtained largely from the literature or calculations from contextual indicators (e.g., body location) or morphological components (e.g., diameter). The latter could include, for example, estimates of typical ranges in lengths multiplied by ranges in cross-sectional area to obtain a mass range for a neuron or myocyte group. The cell-size ranges are shown as horizontal lines in Fig. 4*A*, and are converted to an estimated SD to compute the CV (SD divided by mean) in Fig. 5*B* and variance (SD squared) in Fig. 5*C*.

We compiled 54 published cell-size distributions from the literature for cell types in all major cell groups, drawn from 30 published sources (Dataset S2). Several of these distributions were for the same cell types, in which case we excluded the less complete distribution (fewer size classes), leaving 30 distributions, as shown in Fig. 4*B* and *SI Appendix*, Fig. S2. Many of these distributions reported size in linear or area units, which were converted to mass. This was usually straightforward, but in cases such as with neurons and myocytes, where only a cross-sectional diameter or area was reported, we assumed that the axon or fiber length was constant for the section reported and scaled the mass according to the variation reported. We set the weighted mean cell size to match the mean size obtained from other sources included in our primary dataset (Dataset S1). Although this may introduce an arithmetic and possibly multiplicative bias into the true mass distribution, it should not alter the near lognormal shape of the size distribution for most cell types.

We also compiled size distribution data for 10 model system cell types commonly used in cell-size research. These cell types include the following: *Arabidopsis* shoot meristem cells (80) (*n* = 1,746); human adenocarcinoma cells (81) (HeLa cell line; *n* = 13,848); fission yeast cells (82) (*n* = 329); mouse lymphocytic leukemia cells (81) (L1210 cell line; *n* = 300); human induced pluripotent stem cells (iPSC) differentiated toward presomitic mesoderm cells (83) (iPSC-derived presomitic mesoderm cells; *n* = 299); haploid and diploid budding yeast cells (CDC28 mutant); human lung fibroblast cells; immortalized human mammary epithelium cells (HMEC) cells; and, human retinal pigment epithelial cells (84). For L1210 cells, human iPSC cells and fission yeast cells, data were based on direct cell mass measurements based on buoyant mass in different density fluids (85). For all other cell types, data were originally volumetric (Coulter counter) and converted to mass using a conversion density of 1.056 g/mL.

To normalize these distributions to the same reference scale so that area under all curves are equal (Fig. 5*A*), we divided each binned proportion by the mean bin width and multiplied by the geometric mean of the respective cell mass associated with each bin.

## Supplementary Material

Appendix 01 (PDF)

Dataset S01 (XLSX)

Dataset S02 (XLSX)

## Data Availability

Excel data are available as *SI Appendix* “Dataset S1” and “Dataset S2”.

## References

[1] I. Conlon, M. Raff, Size control in animal development. Cell **96**, 235–244 (1999). 9988218 10.1016/s0092-8674(00)80563-2

[2] M. B. Ginzberg, R. Kafri, M. Kirschner, On being the right (cell) size. Science **348**, 1245075 (2015). 25977557 10.1126/science.1245075 PMC4533982

[3] E. Zatulovskiy, J. M. Skotheim, On the molecular mechanisms regulating animal cell size homeostasis. Trend Genetics **36**, 360–372 (2020). 10.1016/j.tig.2020.01.011 PMC7162994 32294416

[4] A. Tzur, R. Kafri, V. S. LeBleu, G. Lahav, M. W. Kirschner, Cell growth and size homeostasis in proliferating animal cells. Science **325**, 167–171 (2009). 19589995 10.1126/science.1174294 PMC2905160

[5] J. C. Lui, J. Baron, Mechanisms limiting body growth in mammals. Endocrine Rev. **32**, 422–440 (2011). 21441345 10.1210/er.2011-0001 PMC3365796

[6] V. M. Savage , Scaling of number, size, and metabolic rate of cells with body size in mammals. Proc. Natl. Acad. Sci. U.S.A. **104**, 4718–4723 (2007). 17360590 10.1073/pnas.0611235104 PMC1838666

[7] R. Milo, R. Phillips, Cell Biology by the Numbers (Garland Science, 2015).

[8] J. Lengefeld , Cell size is a determinant of stem cell potential during aging. Sci. Adv. **7**, eabk0271 (2021). 34767451 10.1126/sciadv.abk0271 PMC8589318

[9] V. Kumar, A. K. Abbas, N. Fausto, J. C. Aster, Robbins and Cotran pathologic basis of disease, professional edition e-book (Elsevier Health Sciences, 2014).

[10] C. W. Sandlin , Epithelial cell size dysregulation in human lung adenocarcinoma. PLOS One **17**, e0274091 (2022). 36201559 10.1371/journal.pone.0274091 PMC9536599

[11] U. Smith, Effect of cell size on lipid synthesis by human adipose tissue in vitro. J. Lipid Res. **12**, 65–70 (1971). 4322518

[12] M. Pende , Hypoinsulinaemia, glucose intolerance and diminished β-cell size in S6K1-deficient mice. Nature **408**, 994–997 (2000). 11140689 10.1038/35050135

[13] T. P. Miettinen, M. Björklund, Cellular allometry of mitochondrial functionality establishes the optimal cell size. Dev. Cell **39**, 370–382 (2016). 27720611 10.1016/j.devcel.2016.09.004 PMC5104693

[14] G. E. Neurohr , Excessive cell growth causes cytoplasm dilution and contributes to senescence. Cell **176**, 1083–1097.e18 (2019). 30739799 10.1016/j.cell.2019.01.018 PMC6386581

[15] L. Mu , Mass measurements during lymphocytic leukemia cell polyploidization decouple cell cycle- and cell size-dependent growth. Proc. Natl. Acad. Sci. U.S.A. **117**, 15659–15665 (2020). 32581119 10.1073/pnas.1922197117 PMC7355023

[16] E. Bianconi , An estimation of the number of cells in the human body. Ann. Hum. Biol. **40**, 463–471 (2013). 23829164 10.3109/03014460.2013.807878

[17] R. Sender, S. Fuchs, R. Milo, Revised estimates for the number of human and bacteria cells in the body. PLoS Biol. **14**, e1002533 (2016). 27541692 10.1371/journal.pbio.1002533 PMC4991899

[18] A. Campbell, Synchronization of cell division. Bacteriol. Rev. **21**, 263–272 (1957). 13488884 10.1128/br.21.4.263-272.1957 PMC180914

[19] D. O. Morgan, The cell cycle: Principles of control (New Science Press, 2007).

[20] J. F. Gillooly, A. Hein, R. Damiani, Nuclear DNA content varies with cell size across human cell types. Cold Spring Harb Perspect Biol. **7**, a019091 (2015). 26134319 10.1101/cshperspect.a019091 PMC4484964

[21] O. Rozenblatt-Rosen, M. J. Stubbington, A. Regev, S. A. Teichmann, The human cell atlas: From vision to reality. Nature **550**, 451–453 (2017). 29072289 10.1038/550451a

[22] D. Osumi-Sutherland , Cell type ontologies of the human cell atlas. Nat. Cell Biol. **23**, 1129–1135 (2021). 34750578 10.1038/s41556-021-00787-7

[23] E. Mereu , Benchmarking single-cell RNA-sequencing protocols for cell atlas projects. Nat. Biotechnol. **38**, 747–755 (2020). 32518403 10.1038/s41587-020-0469-4

[24] W. S. Snyder , ICRP Publication 23: Report of the Task Group on Reference Man: A Report Prepared by a Task Group of Committee 2 of the International Commission on Radiological Protection (Pergamon Oxford, 1975).

[25] J. Valentin, ICRP Publication 89: Basic anatomical and physiological data for use in radiological protection: Reference values (Elsevier Health Sciences, 2003). 14506981

[26] H.-G. Menzel, C. Clement, P. DeLuca, ICRP Publication 110. Realistic reference phantoms: An ICRP/ICRU joint effort. A report of adult reference computational phantoms. Ann. ICRP **39**, 1–164 (2009). 10.1016/j.icrp.2009.09.001 19897132

[27] B. Alberts , Molecular Biology of the Cell (Garland Science, ed. 3, 1994).

[28] M. K. Vickaryous, B. K. Hall, Human cell type diversity, evolution, development, and classification with special reference to cells derived from the neural crest. Biol. Rev. Camb Philos. Soc. **81**, 425–455 (2006). 16790079 10.1017/S1464793106007068

[29] J. Jo , Hypertrophy and/or hyperplasia: Dynamics of adipose tissue growth. PLoS Comput. Biol. **5**, e1000324 (2009). 19325873 10.1371/journal.pcbi.1000324 PMC2653640

[30] A. C. Paul, N. Rosenthal, Different modes of hypertrophy in skeletal muscle fibers. J. Cell Biol. **156**, 751–760 (2002). 11839766 10.1083/jcb.200105147 PMC2174086

[31] J.-X. Liu , Myonuclear domain size and myosin isoform expression in muscle fibres from mammals representing a 100 000-fold difference in body size. Exp. Physiol. **94**, 117–129 (2009). 18820003 10.1113/expphysiol.2008.043877

[32] W. F. Marshall , What determines cell size? BMC Biol. **10**, 1–22 (2012). 23241366 10.1186/1741-7007-10-101 PMC3522064

[33] Y. Arata, H. Takagi, Y. Sako, H. Sawa, Power law relationship between cell cycle duration and cell volume in the early embryonic development of *Caenorhabditis elegans*. Front. Physiol. **5**, 529 (2015). 25674063 10.3389/fphys.2014.00529 PMC4309120

[34] G. Fankhauser, The effects of changes in chromosome number on amphibian development. Q. Rev. Biol. **20**, 20–78 (1945).

[35] T. P. Neufeld, A. F. A. de la Cruz, L. A. Johnston, B. A. Edgar, Coordination of growth and cell division in the Drosophila wing. Cell **93**, 1183–1193 (1998). 9657151 10.1016/s0092-8674(00)81462-2

[36] C. C. Henery, M. H. Kaufman, Relationship between cell size and nuclear volume in nucleated red blood cells of developmentally matched diploid and tetraploid mouse embryos. J. Exp. Zool. **261**, 472–478 (1992). 1569414 10.1002/jez.1402610414

[37] M. K. Diril , Cyclin-dependent kinase 1 (Cdk1) is essential for cell division and suppression of DNA re-replication but not for liver regeneration. Proc. Natl. Acad. Sci. U.S.A. **109**, 3826–3831 (2012). 22355113 10.1073/pnas.1115201109 PMC3309725

[38] Y. Miyaoka , Hypertrophy and unconventional cell division of hepatocytes underlie liver regeneration. Curr. Biol. **22**, 1166–1175 (2012). 22658593 10.1016/j.cub.2012.05.016

[39] G. K. Michalopoulos, Hepatostat: Liver regeneration and normal liver tissue maintenance. Hepatology **65**, 1384–1392 (2017). 27997988 10.1002/hep.28988

[40] J. W. Prothero, The Design of Mammals (Cambridge University Press, 2015).

[41] X. Gabaix, Zipf’s law for cities: An explanation. Q. J. Econ. **114**, 739–767 (1999).

[42] M. Newman, Power laws, Pareto distributions and Zipf’s law. Contemporary Phys. **46**, 323–351 (2005).

[43] D. W. Hughes, N. W. Harris, The distribution of asteroid sizes and its significance. Planet. Space Sci. **42**, 291–295 (1994).

[44] I. A. Hatton, R. F. Heneghan, Y. M. Bar-On, E. D. Galbraith, The global ocean size spectrum from bacteria to whales. Sci. Adv. **7**, eabh3732 (2021). 34757796 10.1126/sciadv.abh3732 PMC8580314

[45] A. Giometto, F. Altermatt, F. Carrara, A. Maritan, A. Rinaldo, Scaling body size fluctuations. Proc. Natl. Acad. Sci. U.S.A. **110**, 4646–4650 (2013). 23487793 10.1073/pnas.1301552110 PMC3607007

[46] C. James, S. Azaele, A. Maritan, F. Simini, Zipf’s and Taylor’s laws. Phys. Rev. E **98**, 032408 (2018).

[47] L. R. Taylor, Aggregation, variance and the mean. Nature **189**, 732–735 (1961).

[48] Z. Eisler, I. Bartos, J. Kertesz, Fluctuation scaling in complex systems: Taylor’s law and beyond. Adv. Phys. **57**, 89–142 (2008).

[49] R. B. Azevedo, A. M. Leroi, A power law for cells. Proc. Natl. Acad. Sci. U.S.A. **98**, 5699–5704 (2001). 11331756 10.1073/pnas.091485998 PMC33276

[50] J. Ramsayer, S. Fellous, J. E. Cohen, M. E. Hochberg, Taylor’s law holds in experimental bacterial populations but competition does not influence the slope. Biol. Lett. **8**, 316–319 (2012). 22072282 10.1098/rsbl.2011.0895 PMC3297404

[51] H. A. Simon, On a class of skew distribution functions. Biometrika **42**, 425–440 (1955).

[52] G. I. Bell, E. C. Anderson, Cell growth and division: I. a mathematical model with applications to cell volume distributions in mammalian suspension cultures. Biophys. J. **7**, 329–351 (1967). 6069910 10.1016/S0006-3495(67)86592-5 PMC1368064

[53] A. A. Amodeo, J. M. Skotheim, Cell-size control. Cold Spring Harbor Perspect. Biol. **8**, a019083 (2016). 10.1101/cshperspect.a019083 PMC4744813 26254313

[54] D. Huang, T. Lo, H. Merrikh, P. A. Wiggins, Characterizing stochastic cell-cycle dynamics in exponential growth. Phys. Rev. E **105**, 014420 (2022). 35193317 10.1103/PhysRevE.105.014420 PMC9506121

[55] S. Iyer-Biswas , Scaling laws governing stochastic growth and division of single bacterial cells. Proc. Natl. Acad. Sci. U.S.A. **111**, 15912–15917 (2014). 25349411 10.1073/pnas.1403232111 PMC4234605

[56] F. Trepel, Number and distribution of lymphocytes in man. A critical analysis. Klin Wochenschr **52**, 511–515 (1974). 4853392 10.1007/BF01468720

[57] J. Westermann, R. Pabst, Distribution of lymphocyte subsets and natural killer cells in the human body. Clin. Invest. **70**, 539–544 (1992). 10.1007/BF00184787 1392422

[58] K. S. Blum, R. Pabst, Lymphocyte numbers and subsets in the human blood. Do they mirror the situation in all organs? Immunol. Lett. **108**, 45–51 (2007). 17129612 10.1016/j.imlet.2006.10.009

[59] V. V. Ganusov, R. J. De Boer, Do most lymphocytes in humans really reside in the gut? Trend Immunol. **28**, 514–518 (2007). 10.1016/j.it.2007.08.009 17964854

[60] B. Asquith, J. A. Borghans, V. V. Ganusov, D. C. Macallan, Lymphocyte kinetics in health and disease. Trend Immunol. **30**, 182–189 (2009). 10.1016/j.it.2009.01.003 19286425

[61] D. Lambrechts , Phenotype molding of stromal cells in the lung tumor microenvironment. Nat. Med. **24**, 1277–1289 (2018). 29988129 10.1038/s41591-018-0096-5

[62] R. H. Peters, The Ecological Implications of Body Size (Cambridge University Press, ed. 1, 1983).

[63] I. A. Hatton, A. P. Dobson, D. Storch, E. D. Galbraith, M. Loreau, Linking scaling laws across eukaryotes. Proc. Natl. Acad. Sci. U.S.A. **116**, 21616–21622 (2019). 31591216 10.1073/pnas.1900492116 PMC6815163

[64] J. P. Greer , Wintrobe’s Clinical Hematology (Lippincott Williams & Wilkins, ed. 11, 2004).

[65] K. Kaushansky, M. A. Lichtman, E. Beutler, Williams Hematology (McGraw-Hill Medical, ed. 8, 2010).

[66] G. P. Downey , Retention of leukocytes in capillaries: Role of cell size and deformability. J. Appl. Physiol. **69**, 1767–1778 (1990). 2272970 10.1152/jappl.1990.69.5.1767

[67] M. Di Mascio , Noninvasive in vivo imaging of CD4 cells in simian-human immunodeficiency virus (SHIV)–infected nonhuman primates. Blood **114**, 328–337 (2009). 19417212 10.1182/blood-2008-12-192203 PMC2714208

[68] H. W. Vliegen, A. Van der Laarse, C. J. Cornelisse, F. Eulderink, Myocardial changes in pressure overload-induced left ventricular hypertrophy: A study on tissue composition, polyploidization and multinucleation. Eur. Heart J. **12**, 488–494 (1991). 1829680 10.1093/oxfordjournals.eurheartj.a059928

[69] C.-P. Adler, H. Friedburg, G. W. Herget, M. Neuburger, H. Schwalb, Variability of cardiomyocyte DNA content, ploidy level and nuclear number in mammalian hearts. Virchows Archiv. **429**, 159–164 (1996). 8917717 10.1007/BF00192438

[70] M. Mollova , Cardiomyocyte proliferation contributes to heart growth in young humans. Proc. Natl. Acad. Sci. U.S.A. **110**, 1446–1451 (2013). 23302686 10.1073/pnas.1214608110 PMC3557060

[71] Y. Guo, W. T. Pu, Cardiomyocyte maturation: New phase in development. Circulation Res. **126**, 1086–1106 (2020). 32271675 10.1161/CIRCRESAHA.119.315862 PMC7199445

[72] O. Bergmann , Dynamics of cell generation and turnover in the human heart. Cell **161**, 1566–1575 (2015). 26073943 10.1016/j.cell.2015.05.026

[73] F. A. C. Azevedo , Equal numbers of neuronal and nonneuronal cells make the human brain an isometrically scaled-up primate brain. J. Comp. Neurol. **513**, 532–541 (2009). 19226510 10.1002/cne.21974

[74] J. Bahney, C. S. von Bartheld, The cellular composition and glia–neuron ratio in the spinal cord of a human and a nonhuman primate: Comparison with other species and brain regions. Anat. Record **301**, 697–710 (2018). 10.1002/ar.23728 PMC5845477 29150977

[75] H. H. Mitchell, T. S. Hamilton, F. R. Steggerda, H. W. Bean, The chemical composition of the adult human body and its bearing on the biochemistry of growth. J. Biol. Chem. **158**, 625–637 (1945).

[76] R. Mlcoo, A. R. Forbes, H. H. Cooper, Mitchell, The composition of the adult human body as determined by chemical analysis. J. Biol. Chem. **203**, 359–366 (1953). 13069519

[77] R. M. Forbes, H. H. Mitchell, A. R. Cooper, Further studies on the gross composition and mineral elements of the adult human body. J. Biol. Chem. **223**, 969–975 (1956). 13385244

[78] K. Hosoda, T. Matsuura, H. Suzuki, T. Yomo, Origin of lognormal-like distributions with a common width in a growth and division process. Phys. Rev. E **83**, 031118 (2011). 10.1103/PhysRevE.83.031118 21517465

[79] R. Hanel, B. Corominas-Murtra, B. Liu, S. Thurner, Fitting power-laws in empirical data with estimators that work for all exponents. PloS One **12**, e0170920 (2017). 28245249 10.1371/journal.pone.0170920 PMC5330461

[80] A. Serrano-Mislata, K. Schiessl, R. Sablowski, Active control of cell size generates spatial detail during plant organogenesis. Curr. Biol. **25**, 2991–2996 (2015). 26526374 10.1016/j.cub.2015.10.008 PMC4651904

[81] J. H. Kang , Noninvasive monitoring of single-cell mechanics by acoustic scattering. Nat. Methods **16**, 263–269 (2019). 30742041 10.1038/s41592-019-0326-x PMC6420125

[82] P. D. Odermatt , Variations of intracellular density during the cell cycle arise from tip-growth regulation in fission yeast. ELife **10**, e64901 (2021). 34100714 10.7554/eLife.64901 PMC8221806

[83] M. Diaz-Cuadros Metabolic regulation of species-specific developmental rates. bioRxiv [Preprint] (2021). 10.1101/2021.08.27.457974 (Accessed 30 August 2021).

[84] M. C. Lanz , Increasing cell size remodels the proteome and promotes senescence. Mol. Cell **82**, 3255–3269.e8 (2022). 35987199 10.1016/j.molcel.2022.07.017 PMC9444988

[85] W. H. Grover , Measuring single-cell density. Proc. Natl. Acad. Sci. U.S.A. **108**, 10992–10996 (2011). 21690360 10.1073/pnas.1104651108 PMC3131325

