# Supplementary material for: The human cell count and size distribution
Source: Proc Natl Acad Sci U S A. 2023 Sep 18;120(39):e2303077120. doi: 10.1073/pnas.2303077120 (PMC10523466; doi:10.1073/pnas.2303077120)
Supplement: Supplementary file 1 — Appendix 01 (PDF) [file pnas.2303077120.sapp.pdf]

PNAS

Supporting Information for
The human cell count and size distribution

Ian A. Hatton, Eric D. Galbraith, Nono Saha Cyrille Merleau, Teemu P. Miettinen, Benjamin McDonald Smith & Jeffery A. Shander

Corresponding author: Ian Hatton
Email: i.a.hatton@gmail.com

This PDF file includes:

Supplementary Methods
Supplementary Text
Figures S1 to S9
References

Other supporting materials for this manuscript include the following:

Dataset S1.xlsx
Dataset S2.xlsx
https://humancelltreemap.mis.mpg.de/

TABLE OF CONTENTS

**Supplementary Methods**	3
- Blood cells	3
- Myocytes	4
- Epithelial & endothelial	5
- Stem, germ & pericyte	6
- Adipocytes	6
- Neuron & glial	7
- Fibroblast & osteoid	9
**Supplementary Text**	10
- Outstanding uncertainties	10
- Differences with prior whole-body cell counts	10
- Notes to figures	11
**Supplementary Figures**	13
Fig. S1. Cell mass vs. body mass scaling across mammal species.	13
Fig. S2. Published cell size distributions.	14
Fig. S3. Constructing the size frequency histogram.	15
Fig. S4. Alternative characterizations of the cell size distribution.	16
Fig. S5. Cell size-distributions across male, female and child models.	17
Fig. S6. Cell size variation across the human cell size range.	18
Fig. S7. Treemaps by major cell group.	19
Fig. S8. Treemaps by organ systems.	20
Fig. S9. Additional tree maps of interest.	21
**References**	22

SUPPLEMENTARY METHODS

We summarize our methods in more detail from those listed in the main paper, highlighting key uncertainties for each major cell group. All data along with more detailed notes and methods are available as supplementary datasets (Dataset S1.xlsx, Dataset S2.xlsx) and available through https://humancelltreemap.mis.mpg.de/.

Although we describe our methods according to the seven broad cell classes used in the paper, cell count data were estimated according to separate tissues and components. Our data are organized according to separate classifications that include both the organ-tissue and the cell class-type hierarchies. We focus on the reference male model, unless otherwise stated.

Blood cells

The reference male contains 4.7 kg of blood cellular mass, (55% intravascular, 45% extravascular tissue-resident). Roughly half of the tissue resident cell mass resides in the four blood organs (Fig. 6). We have classified blood cells into n=53 blood cell types, and enumerated many of these across some 30 tissue systems. In total we consider 191 blood cell groups, which includes broad cell types in each major tissue, much of the data for which derives from Refs: (1–4). Our total nucleated blood cell count of 3.8 trillion consists of ≈2.1 trillion lymphoid cells, ≈1.1 trillion granulocytes, ≈0.4 trillion erythrocyte precursors, ≈0.21 trillion macrophages, and ≈0.024 trillion monocytes.

The commonly measured complete blood counts (CBC) provide standard estimates of the size and number of cells in the circulating blood. Intravascular blood cells are thus straightforward to estimate given the reference male blood volume of 5.2 L (Ref: (5, 6)). Non-nucleated cells account for more than 99% of the intravascular blood cells (≈27 trillion) and some ≈2 trillion in the spleen and red bone marrow. Blood cell diameters derived from uncorrected blood-smears were corrected downward by 30% to 50% to estimate the actual in-suspension diameter. Bone marrow cells are commonly measured via bone marrow biopsies and aspiration (1, 2), allowing estimates of a total bone marrow nucleated cell count of $\approx 1.4\times10^{12}$, of which ≈56% are in the granulocyte lineage, ≈27% are in the erythroid lineage, ≈15% are estimated to be lymphocytes, and ≈2% address monocytes, macrophages and other small lineages (1, 2).

The nucleated cells and particularly the various kinds of lymphocytes in the remaining blood organs and other tissues are less well estimated, and our attempts to consolidate relevant published data turned up several large discrepancies with prior work (7–9) (see Fig. 6). Our lymphocyte count in the lymph nodes ($\approx 1.1\times10^{12}$) and spleen ($\approx 2.9\times10^{11}$) derive from lymphocyte counts per gram of primate tissue (rhesus monkey) measured in both organs (4) and extrapolated to human tissues. There are an estimated additional $\approx 8.5\times10^{10}$ lymphocytes in the thymus. These estimates yield a combined lymphocyte count across the major blood organs of ≈1.7 trillion. Combined with lymphocyte estimates over some 30 remaining tissues, we estimate the total count is approximately 2 trillion lymphocytes.

Our estimated lymphocyte count is approximately four fold higher than prior estimates (7–9). These prior counts are based, in part, on extrapolations from rodent DNA concentrations for the spleen and lymph nodes, from which lymphocyte proportion and size were further estimated to extrapolate to human spleen and lymph nodes (9). These rodent-derived counts yield values approximately one fifth of the more recent primate-derived values (4). Underestimates of a similar order are also evident in these studies with respect to the bone marrow nucleated cell count (7–9), which are approximately one half the value of more recent clinical measurements (1, 2). Other sources of uncertainty relate to the fact that cytometric optical vs. impedance vs. microscopy-based histological measurements can often give different estimates of size and count (1–3). Our estimates are based on more closely related specimens with arguably fewer assumptions than prior work, but we emphasize that improving the quantification of nucleated blood cells, particularly lymphocytes, should be a focus of future work.

Myocytes

Myocytes include the striated skeletal myocytes and cardiomyocytes, as well as the smooth muscle cells distributed throughout the body, such as in the gastrointestinal and uro-genital tracts.

The skeletal muscles in the reference male are 28 kg, approximately 6 kg of which is extracellular water and extracellular protein, and 500 g of which are tissue stromal cell mass, leaving slightly less than 22 kg of myocyte mass. In contrast, skeletal muscles in the reference female are 17.5 kg and in the reference child of 10 years, only 11 kg, constituting the largest tissue biomass differences between reference models. Following observed modes of hypertrophy in skeletal myocytes (10), we assumed that myocyte counts are largely fixed across reference models. We thus scaled myocyte size based on the ratios of the total muscle mass of female (0.625) or child (0.393) to male.

Given how widely skeletal myocytes vary in size, from the stapedius in the inner ear ($\approx 3\times10^{-7}$ g) to the sartorius in the thigh ($\approx 7\times10^{-4}$ g), we compiled separate myocyte mean sizes and counts for 243 skeletal muscle groups in the body, addressing 350 muscle pairs (assuming right and left side are equivalent). Eight of the 243 groups account for 72 small muscle pairs primarily associated with the vertebral column and rib cage, such as the deep intrinsic muscles of the back and spine. Another three groups are aggregate estimates of fiber size and count for 46 muscle pairs, which had insufficient data for estimating specific muscle fiber size and count.

The primary dataset (Dataset S1.xlsx) includes a skeletal myocyte estimate for each muscle group and provides brief notes on how these were calculated. Additional standard muscle parameters, including length, volume, pennation angle and cross-sectional area for both the muscle group and its component fibers (myocytes) are not always listed if they are not directly relevant to our

calculation. The dataset includes a record ID number for each skeletal muscle group that is retained in our dataset to facilitate future recall of these parameters.

Cardiomyocytes are estimated in the reference male heart to be 330 g, of which 90 g is extracellular mass, and total cellular mass is 240 g (without blood). Cardiomyocyte counts are based on published counts of myocytes for specific sections of the heart (11–13), (e.g. left ventricle, or both ventricles, etc.), that were then extrapolated to the entire heart and averaged over all studies. Obtaining values for cardiomyocytes in the reference female and child models made use of reported patterns of cardiomyocyte maturation and hypertrophy (14, 15) to roughly scale both fiber size and count to heart mass in each model.

Smooth muscle is distributed throughout the body (n=27 cell types and n=41 cell groups), predominantly in the gastrointestinal and uro-genital tracts, but also in the various exocrine glands, skin hair follicles, tissues of the head, trachea, large blood vessels etc. Estimates of cell size and count in each body tissue made use of specific studies, where available, or else generic myocyte sizes. Counts were often calculated from reported myocyte fractions relative to other cell types, for which we have better estimates. Cell counts were scaled to the relevant tissue mass in which they reside (17, 18), and across reference male, female and child models (5, 6, 16).

Epithelial & endothelial

Epithelial and endothelial cells provide the outer covering and inner lining of most of the body's tissue systems. Epithelial cells also make up the majority of the well-known cell types in the body (n=142 unique cell types and 228 cell groups), and more specific methods are needed than can be covered here. Even within a cell type, cells may have different sizes and shapes depending on their position (e.g. the epidermal cells of the skin are cuboid in the basal layer and squamous or flattened in the stratum corneum). While some cell types, such as hepatocyte size and proportion within the liver, are well studied, other cell types, such as the sebocytes of the exocrine glands of the hair follicles, are distributed throughout the body at different densities, and require multiple calculations in different locations.

For many tissues, estimates were made of cell count using a generic cell apical surface area and a total internal and external tissue surface area. Epithelial and endothelial cells cover the majority of an estimated 1000 to 1200 m^2 of total tissue surface area in a single layer of simple cuboid, columnar or squamous cells. Numerous estimates of surface area for different tissue systems and body locations can thus be used to estimate the number of epithelial and endothelial cells throughout the body. These were cross checked against associated biomass estimates and "ground-truthed" with specific studies on particular systems. For example, the whole capillary system is thought to sum to a total length of as much as 19,000 km (19), which can be used with a mean capillary diameter (7 to 8 μm) to estimate total capillary surface area (≈480 m^2). Where possible, microvascular studies providing capillary densities (mm capillary per mm^3 of tissue) in different locations were used in conjunction with endothelial apical surface area (600 to 1350 μm^2), to put

bounds on endothelial cell counts for different parts of the body. Similar calculations were used to estimate the endothelial cells of the lymphatic capillaries, which are estimated to be ⅕ the length of those of the cardiovascular system (19), along with a mean lymphatic capillary diameter (≈12 to 20 μm).

Numerous additional calculations were undertaken for the various kinds of glands, ducts, follicles, tubules, islets, acini and other sub-tissues making our estimates of epithelial and endothelial cells among the most extensive components of the dataset.

Stem, germ & pericyte

Stem cells are undifferentiated cells with the capacity to proliferate and differentiate into more specialized cells. Our data include n=39 stem cell types distributed across most major body systems (73 stem cell groups). In the absence of location specific estimates of stem cell size, we used a generic cell size, and determined counts in various tissues based primarily on ratios of stem to other cell types in each tissue.

We refer to germ cells as all potentially reproductive cells in the germline, thus including haploid gamete cells. The reference male germ cells thus refer to the various diploid and haploid stages of sperm formation (n=13 cell groups), while the reference female germ cells refer to the various groups of oocytes (n = 10 cell groups). The majority of cell mass and count estimates derive directly from the literature, and represent mean values, where multiple estimates are available.

Pericytes wrap around the endothelial cells of the capillaries, and are known to have a variety of functions. Our data estimate pericyte counts in 38 tissues based primarily on location-specific pericytes to endothelial cell ratios, the latter of which we have estimated separately (see previous sub-section).

Adipocytes

The reference male model adipose tissue accounts for 15 to 18.2 kg of total mass, depending on whether one uses a 70 kg (5) or a 73 kg (6, 16) ICRP Reference man. We use the former, where adipose tissue accounts for 15 kg of 70 kg total mass, 12 kg of which is stored fat mass, though primary sources exhibit wide variability (5). The reference male potassium-based total cellular mass of adipocytes is 13.3 kg (5), which is distributed as hypodermal (≈6.5 kg), yellow bone marrow (≈2.2 kg), visceral (≈3.5 kg), and the interstitial adipose tissue in different organs (≈840 g), with an additional 300 g of stromal cell mass, partitioned proportionately across the four groups. We summarize the size distribution of white adipocytes into four broad size classes in three tissues (hypodermal, visceral and yellow bone marrow) and a single size class in the interstitial tissue of eight organ systems. These values differ significantly in female and child models, totaling 18.1 kg and 6.9 kg of adipocyte cellular mass respectively (6). We assumed that the relative proportions of

different cell masses of adipocytes were constant across all anatomical models and scaled the adipocyte counts according to the relative adipose tissue masses (6).

Although the adipocyte size distribution is well studied for both visceral and subcutaneous, it is more commonly reported for people overweight or obese, and thus in some respects not representative of a reference human model. There also exists uncertainty in the left tail of the size distribution (small adipocytes) that appears to depend on the methods used for size determination (20), but which typically exhibits an additional mode or continual increase in number at smaller sizes. This left tail may or may not indicate the presence of brown adipocytes in visceral adipose tissue whose size (≈5.4 ng) is much smaller than the mean size of white adipocytes, differing by two orders of magnitude. Brown adipose tissue (≈870 g), with a total adipocyte cellular mass of 760 g is allocated to the visceral adipose distributed mostly around the spinal vertebrae, abdominal viscera, neck and upper torso (21, 22).

Further research is needed given some uncertainties in the adipocyte size distribution. Not only is the size distribution unusually broad (Fig. S2; light blue) (20), but most size distributions are from subjects that are reportedly overweight, and there are methodological questions related to the left tail of the size distribution (20).

Neuron & glial

Neuron and glial cell values require a variety of methods and pose many challenges. In total, our data lists mean cell masses and cell counts for 57 neuron cell types (159 groups) and 22 glial cell types (81 groups) in various locations of the central and peripheral nervous systems, as well as other tissue systems, particularly the enteric nervous system of the gastrointestinal tract.

The brain of the reference male has a mass of 1450 g (Ref: (6)), ≈1080 g of which is cellular mass, distributed into the cerebrum and cerebral cortex (≈890 g), the cerebellum (≈110 g) and the brain stem and rest of brain (≈80 g). By taking published ratios of white matter vs. grey matter, as well as reported distributions of axons, glial cells, neurons and stromal cells, in each section of the brain, we undertook cross checks on the various counts, as well as the relevant mean cell masses in various brain components. We estimate a total of 88 billion neurons and 87 billion non-neurons, consisting of ≈53 billion glial cells, ≈4 billion microglial macrophages, and 30 billion other stromal cells in the brain of the reference male. This is consistent with a 1:1 neuron to non-neuron ratio and a total brain cell count of 170 ± 13 billion (Ref: (23)). The ≈30 billion stromal cell count, which has sometimes been previously ignored, is comprised of the vascular network (pericytes and endothelial cells) and their estimated counts are consistent with studies of the CNS microvascular (24–26) and pericyte associated blood-brain barrier (27–29).

The peripheral nervous system (PNS) does not have a listed mass in ICRP documents (5, 6, 16), and is here estimated at 300 g from autopsies (30–32), 10 to 30% of which is nerve-fiber (neurons and associated Schwann cells), and perineural matrix (33). The remainder is associated with

extracellular matrix and epineural related stromal cell mass, including adipocytes. The size of the insulating Schwann cells depends largely on neuron axon diameter. Myelin thickness is estimated by subtracting axon diameters from nerve fiber diameter, or using reported g-ratios for different classes of neurons. The count of satellite glial cells encasing neuron somas in the peripheral ganglia are determined from soma size and counts in the ganglia. To determine specific cell sizes and counts, the PNS and spine are partitioned into different locations and functional categories, as follows:

- body location (torso, lower limbs, upper limbs, etc.);
- major ganglia categories (ventral spine, dorsal root, paravertebral, prevertebral, brainstem, and cranial);
- major motor nuclei (spinal cord ventral root nuclei and brainstem nuclei of the cranial nerves);
- PNS subdivisions (somatic, autonomic, enteric);
- neuron functional categories (afferent versus efferent; and afferent sub-groups of pain, proprioception, temperature or touch).

We estimate the spinal cord count at ≈210 million neurons, with 1.1 billion glial cells (34), the PNS at 59 million neurons with 5.3 billion glial cells, and the enteric nervous system at 0.4 billion neurons with 2 billion glial cells. In addition, we provide estimates for vascular cells, perineural epithelial cells, cranial optic nerve microglia, and epineural adipocytes, as well as other stromal cells.

There are several challenges involved in estimating the size and number of neurons and glial cells. Inconsistencies are often found in the classification of neuron and glial cell types between sources (35–37). There are also widely varying ratios of neurons to glial cells, not only among locations of the nervous system, but also across different studies and subjects. Moreover, estimating mean cell mass for each cell type is necessarily rough, derived from calculations based on neuron or glial cell morphologies which can be highly variable. For example: central nervous system (CNS) glial cells are stellate in form, with variable stellar protrusion lengths; peripheral nervous system (PNS) Schwann cell sizes are axon-diameter dependent; and, most neurons have variable axon and dendrite lengths and diameters. Neuron and glial cell size estimates are thus prone to greater uncertainty than most other cell types.

These challenges are particularly pronounced in the peripheral nervous system (PNS), which extends throughout the majority of tissues in the body, and where cell size and count vary over several orders of magnitude. Axon lengths range from sub-millimeter for nerves in the eyes, up to 1.5 m for some lower limb nerves (e.g. proprioceptive pseudo-unipolar neurons), and axon diameters range from <0.5 μm (e.g. cranial neurons) up to 15 μm (e.g. somatomotor and somatosensory neurons). Adding to this uncertainty, PNS nerve-root axon counts are often based on light microscopy, and are incapable of accurate counts for unmyelinated sub-micron diameter

axons (38). Attempts to estimate unmyelinated peripheral neurons can signifcantly alter the total count, with different studies offering widely different estimates (38).

For the reference female and child models we assumed that the masses of individual neuron cells in the CNS and the masses of glial cells in both the CNS and PNS were constant across male, female and child models. We then scaled the counts of these cells by the tissue mass ratios of the various CNS and PNS tissue components (6, 30–32), such as the ratios of female to male cerebral cortex (1065 g / 1188 g = 0.9), cerebellum (133 g / 148 g = 0.9), or components of the PNS. However, for neurons in the PNS, we assumed that cell counts were approximately constant across anatomical models, and that cell masses could be approximately scaled by the ratios of PNS tissue components.

Fibroblasts & osteoid

Fibroblasts are distributed throughout all tissue systems of the body and synthesize the extracellular matrix and collagen. We use specific values for cell size and count in different tissue systems, where available. Where such data are not available, we use one of several generic fibroblast cell sizes and other reported ratios of fibroblast to other cell types, collagen or other masses, depending on their relevance to a given tissue. We estimate fibroblast cell sizes and counts in 56 tissue systems, which include chondrocytes, tenocytes, keratocytes, reticular cells, etc.

Osteoid cells include the osteoblasts and osteocytes, which form the bone matrix, and odontoblasts and cementocytes in the teeth (osteoclasts are included in the macrophage blood cell category). The various osteoid cell sizes and densities in primary bone were estimated as the averages of several studies. The skeletal mass of the reference male is 10.5 kg, separated into 5.5 kg of bone mass, ≈3.7 kg of bone marrow, and ≈1.3 kg of cartilage and miscellaneous tissue (39). The 5.5 kg of bone mass contains only ≈180 g of cellular mass, ≈104 g of which are osteoid cells (≈0.13 trillion).

Fibroblasts and osteoid cell masses and cell type ratios were assumed constant across reference male, female and child models, while counts were scaled according to relative cellular mass ratios of the relevant tissues.

SUPPLEMENTARY TEXT

Outstanding uncertainties

We highlight cell types and tissues prone to greatest estimation uncertainty. While these uncertainties are not expected to alter our overall findings, they are important for more focused applications.

Our estimates of nucleated blood cell counts in the major blood organs are three to five times higher than prior estimates (7–9). For the total body lymphocytes, in particular, we suggest a four-fold upward revision. However, the dynamic nature of lymphocyte count in different tissues based on health status, and the varying estimates in lymphocyte size based on measurement technique (1–3), as well as their importance for immune function, highlight the need for future research with modern high-precision measurements.

We have also highlighted the challenges in estimating size and counts of neuron and glial cells, and particularly the neurons of the peripheral nervous system. These challenges include varying neuron to glial ratios, the varied classifications of neuron types (35–37), varying morphology and size range throughout the body and the limitations of light microscopy for estimating small unmyelinated axons.

Finally, stromal cells in general and vascular cells in particular create significant challenges. Data supporting accurate lymphatic microvascular density are limited, resulting in potentially wide ranges of endothelial cell counts. Similar issues exist for perivascular cell counts, given the wide range in reported perivascular to endothelial cell ratios (ranging ten-fold). Both cell types are typically small ($< 4\times10^{-10}$ g), and in each tissue are of relatively small biomass, which makes total cell counts prone to error. Small variations in cell size can lead to very different counts, and their small biomass make them less amenable to tissue-mass cross-validation. Nonetheless, due to the pervasiveness of the vasculature throughout the body, these cell types represent a significant cell count and biomass.

Differences with prior whole-body cell counts

Prior studies of total body cell count obtained estimates of 37.2 trillion (Ref: (7)) and 29.6 trillion (Ref: (8)), the latter of which was estimated for a similar 70 kg reference male (8). Our estimate of 36.4 trillion thus differs from the latter study (8) by almost 7 trillion cells. Of this 7 trillion difference, 3 trillion are non-nucleated and 4 trillion are nucleated cells. The major sources of these differences are summarized below.

For the non-nucleated cells, we estimate an additional 2 trillion red blood cells and platelets in the spleen and reticulocytes in the bone marrow, which were not considered in Refs (7, 8). We also estimate an additional ~1 trillion red blood cells in the intravascular, obtained from the ICRP

reference male data of 5.2 L of blood, combined with 5×10^6 red blood cells per μL (5, 6). These ICRP estimates are based on 18 original studies involving 1160 subjects (6)

For the nucleated cells, our estimates differ in three primary areas:

- a higher bone marrow cell count difference of ~0.7 trillion, deriving from bone marrow biopsies and aspiration (1, 2);

- a larger number of lymphocytes in the lymph node, spleen, thymus, and other tissue, summing to a difference of ~1.4 trillion. Our estimate is based on primate lymphocyte counts per gram tissue (4), resulting in a count that is ~4 fold higher than prior estimates based on rodent lymphocyte estimates (9) (see "Blood cells" section, above);

- an additional 2 trillion cells distributed among tissues for which the cell counts were not fully enumerated by Refs: (7, 8). These studies omitted cells in the kidneys (~95% of the nephron and stromal cells), the skin (~3 million sweat glands and ~2 million hair follicles), the heart (cardiomyocytes), the cardiovascular (perivascular cells), and the lymphatic system (endothelial cells). Moreover, significant stromal cell content was omitted for skeletal muscles, adipose tissue and the gastrointestinal tract.

Despite these differences, much of the remainder of our data are largely in agreement with prior counts in most other tissues, and our approach borrows heavily from these pioneering studies (7, 8).

Notes to figures

Fig. 1 represents the cell counts and total cellular biomass in a 70 kg reference male, across different cell types, colored by major cell group. A Voronoi tree (as we are using it here, and following (8)) represents the distribution of different fractions of the whole body as shapes whose relative area is proportional to the relative count or biomass. Tree maps are intermediate between linear and logarithmic axis scales, and better display the differences between cell groups (approximately square root). More specific tree maps are shown in Fig. S7 (classified by cell group) and Fig. S8 (classified by organ system), with additional tree maps of interest in Fig. S9. All Voronoi tree maps were constructed using the d3 javascript library available at https://github.com/Kcnarf/d3-voronoi-treemap, and available in the R library "voronoiTreemap". In panel A, a small area means a small number of cells for that cell type, while in B, it means a small total biomass for that cell type, which could be some combination of small number and/or small cell size. We have removed from each treemap (A & B), the most dominant cell type, since its area would leave little space to see other cell types. Non-nucleated red blood cells and platelets are removed from the Count treemap (A), since they are four times more numerous than all other cells combined. Skeletal myocytes were removed from the Biomass treemap (B), since these muscles represent half the cellular biomass of the body.

Fig. 2 also represents cell counts and cell biomass, broken down across 32 different tissue systems, and again colored by major cell group for a reference male. Another 28 tissue systems are not included in the figure, with the blood cells of the intravascular and major blood organs shown separately in Fig. 6. Note that we only consider the ~40 kg cellular components of this set of 32 tissues. The circulating blood and blood organs add 3.6 kg, and other unaddressed tissues add 0.7 kg, giving a total of ~44.3 kg of cell mass. The remaining mass in the 70 kg male resides in extracellular content, including water (18 – 19 kg), protein (~3.7 kg), and minerals (~3.3 kg, most of which are bone minerals) (5). See the Supplementary "Dataset S1.xlsx" for additional details and references.

Fig. 3 shows how cell size is related to cell number over all cells of the body. We group cell types based on their mean size into logarithmic size classes, with each larger size class twice as large as the adjacent smaller size class. We sum the total counts of each cell type, in each size class, to estimate the bin height. We then fit a least squares regression between bin height and the geometric mean of bin size. Our data, however, only consider the mean cell size for each type, which would introduce irregularities in the overall size distribution, given that we know that cell sizes of a given type often vary lognormally out from the mean (Fig. S2). We can thus spread the counts associated with each mean size lognormally across the typical size range of every given cell type to better approximate the overall size distribution (Fig. S3 provides a schematic of these steps). The small colored dots represent counts associated with a mean size, thus showing the actual data that we assembled in the dataset. In cases where a cell type has a very broad size distribution (e.g. adipocytes, light blue), their counts should spread across multiple size classes. The count of the mean size, therefore, can be higher than the bin sum (e.g. light blue dots have a higher count than their mean-associated size class). Fig. S5 compares the male reference cell size distribution shown in Fig. 3 to those for female and child.

Fig. 4 shows all the same data as Fig. 3, but multiplies the count of each cell type by its mean size to estimate total biomass for each type. In this figure, however, we show the reference female in A and compare it to male and child in B. For those cell types with available size range data, we also plot size range as a horizontal band. This representation clarifies how the counts associated with the mean size of a highly variable group of adipocytes (light blue points) or myocytes (orange points), should spill over into adjacent size classes. Representing the data in this way, also better highlights likely meaningful variation across the cell size range.

Fig. 5 shows the variability in cell size across different cell types. In panel A, we compare previously published datasets reporting the full cell size distribution across 30 human cell types and 10 model system cell types. We find these data are well approximated by a lognormal distribution (Fig. S2 shows 35 of these size distributions in more detail). In panel B and C, we plot the statistics of these 30 human cell distributions as large open circles, while the 10 model system cell types are plotted as black shapes (see Fig. S2 for legend). Overlaid are the cell types from our dataset, where cell size range (if available) is used to estimate CV and variance, and plotted as small colored circles. Panels B and C are different representations of the same cell size variation.

Coefficient of variation (CV in B) is the standard deviation, which is the square root of variance (y in C), divided by the mean cell mass x, $CV = \frac{y^{1/2}}{x}$. If variance scales with cell mass as $y \sim x^k$, (where k = 2.17 in panel C), then $CV \sim x^{\frac{k}{2}-1}$, (where the expected exponent is $\frac{k}{2} - 1 \approx 0.085$), showing the weak dependence of CV on cell size.

Fig. 6 shows the blood cell biomass and count distributions across the intravascular and four major blood organs, colored by major cell group, in a similar manner to Fig. 2. Here we briefly distinguish our methodology from prior work, given some of the large discrepancies that were found in some cell groups, such as lymphocytes in the blood organs, and described in greater detail in the "Blood cells" section.

SUPPLEMENTARY FIGURES

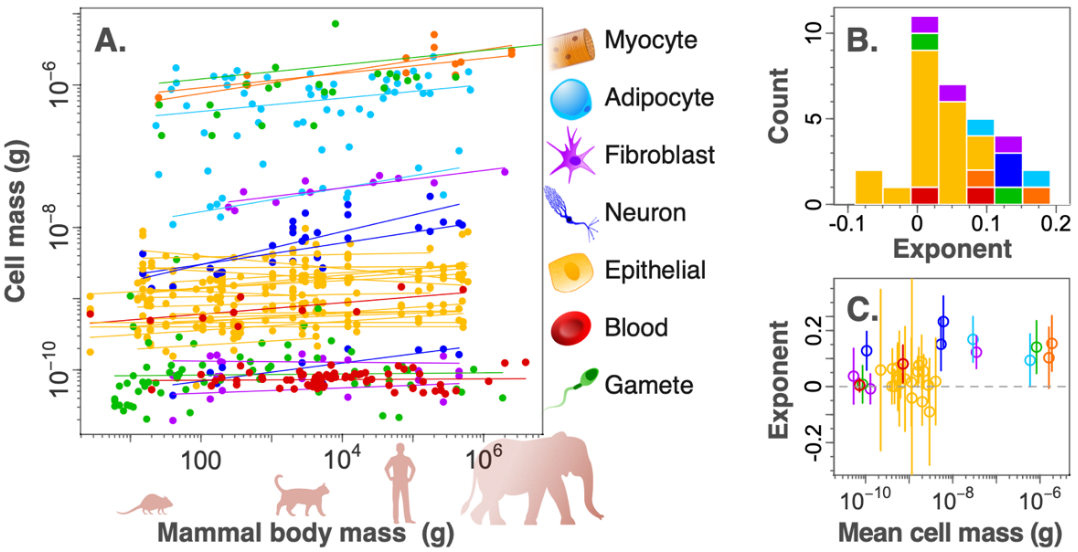

Fig. S1. Cell mass vs. body mass scaling across mammal species. Most major cell types are near invariant with mammal body mass (slope near zero). Exceptions are myocytes, adipocytes and neurons which have positive scaling (all slopes <0.25). Note that the larger violet cell type is an osteocyte (rather than a fibroblast). In total, there are 544 cell measurements across 33 distinct cell types, measured variously over some 170 mammal species. Most cell types are epithelial, but there are at least two cell types in each of our seven major cell classes. The larger green gametes are ova, and there are two monotreme species excluded. **A.** Cell mass vs. body mass for separate cell types measured in different mammal species. **B.** Exponent values (slopes on log-log axes) for the mass-scaling of 32 cells types across mammal species (one neuron type with exponent 0.23 not shown). **C.** The geometric mean cell mass is plotted against the scaling exponent (and 95% CI) for the 33 cell types shown in A. These data are available in Dataset S2.xlsx

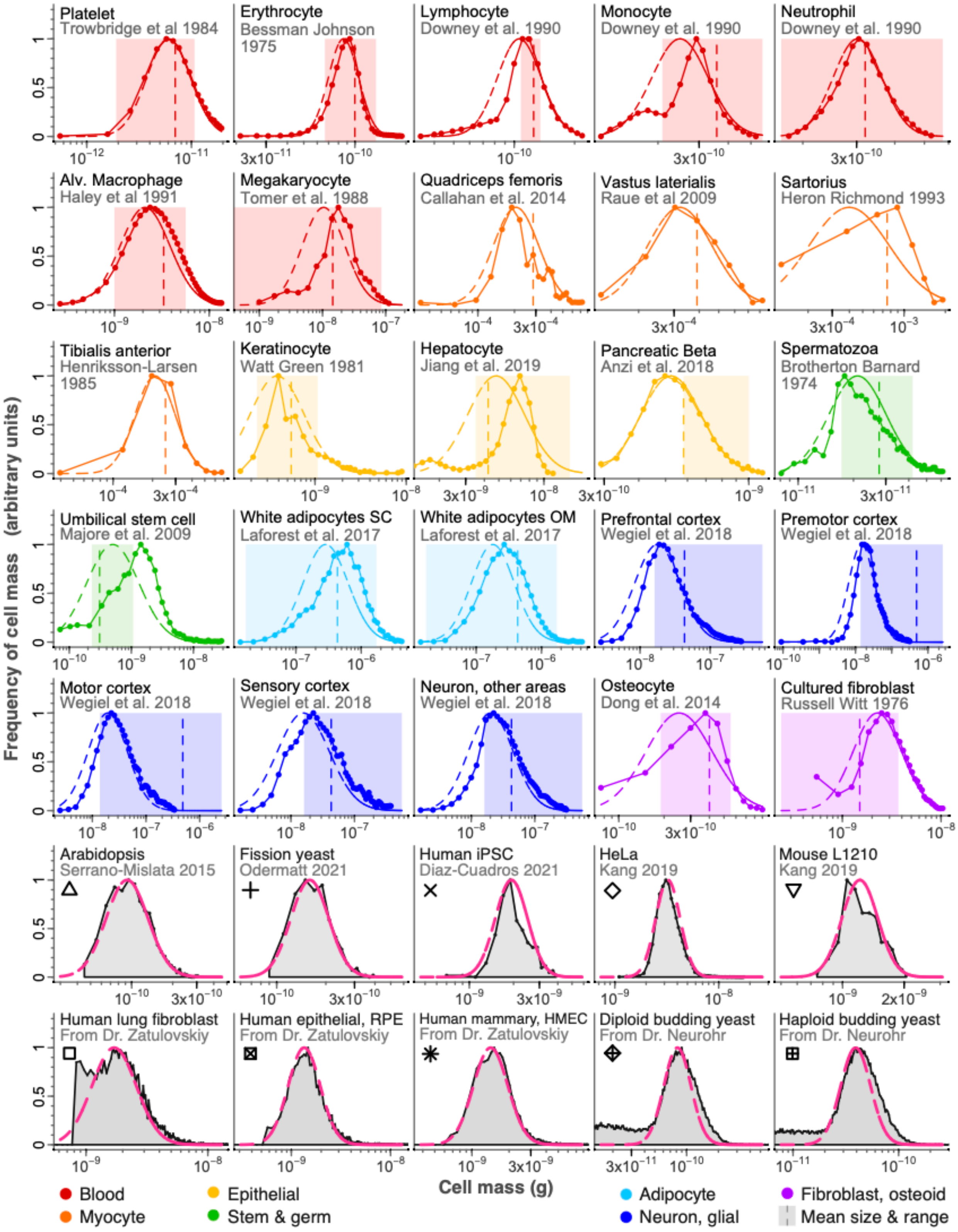

Fig. S2. Published cell size distributions. The empirical size distributions for 25 cell groups and 10 cell size model systems are fit to a lognormal distribution (dashed line), and available in Dataset S2.xlsx. The vertical dashed line and shaded rectangle shows respective mean cell size and range reported in our main dataset, Dataset S1.xlsx . The y-axis has arbitrary units.

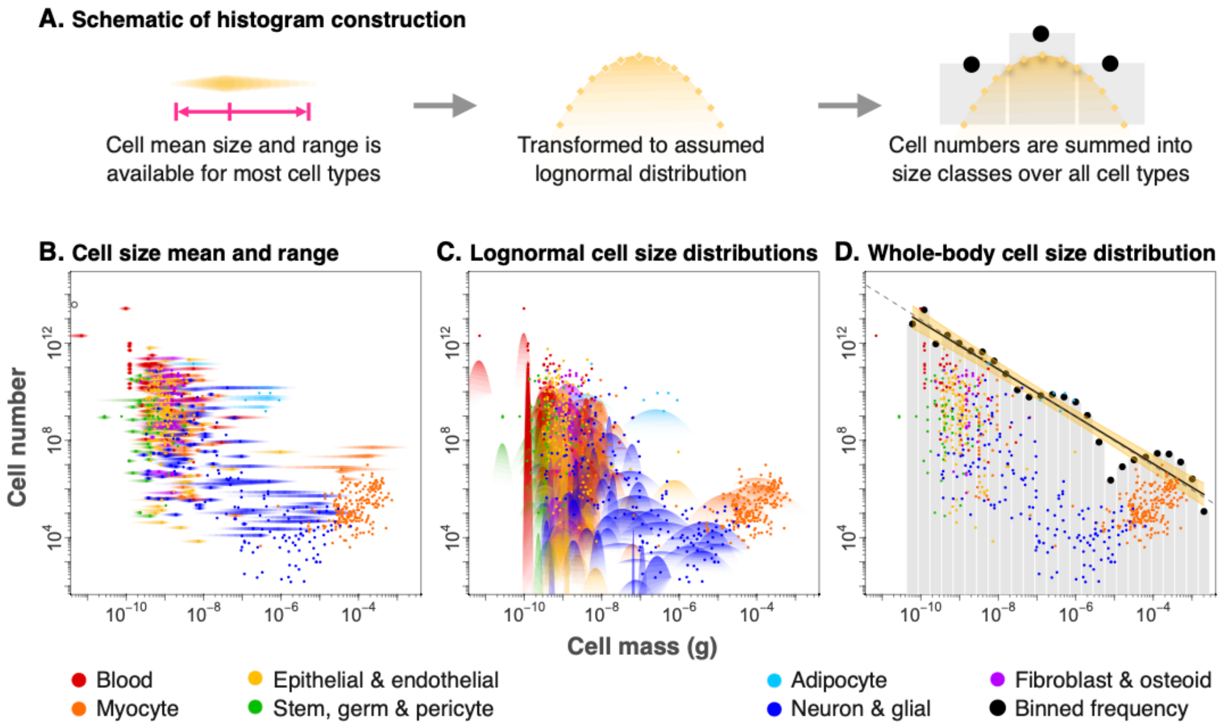

Fig. S3. Constructing the size frequency histogram. A. Schematic of the steps taken to build the cell size distribution, with the relevant data shown directly below in B to D. First, the cell mass mean and range for 1020 (of 1264) cell groups are each transformed into a lognormal distribution, as we assume this represents a reasonable spread of the data about its mean (Fig. S2). Next, each cell group distribution is partitioned into a set of discrete logarithmic size classes. Finally, each size class is summed across all relevant cell groups to build the overall size-frequency histogram. **B.** The mean and range in cell size was estimated for most cell types from prior work. For cell groups for which range data were not available, we used the regression statistics for mean-variance scaling: variance in cell mass = 4.1 × mean cell mass$^{2.17}$ (Fig. 4 D). This equation allows an estimate of standard deviation for any given cell mass, from which a range can be estimated (four standard deviations). **C.** The size range and mean were used to calculate shape and scale parameters for the lognormal distribution for all cell groups, which allowed a count associated with each mean cell mass to be spread across its characteristic size range. **D.** The total count of all cells were then summed into logarithmic size classes to build the size-frequency distribution shown in Fig. 3, as well as the cumulative distribution function shown in Fig. S4 C.

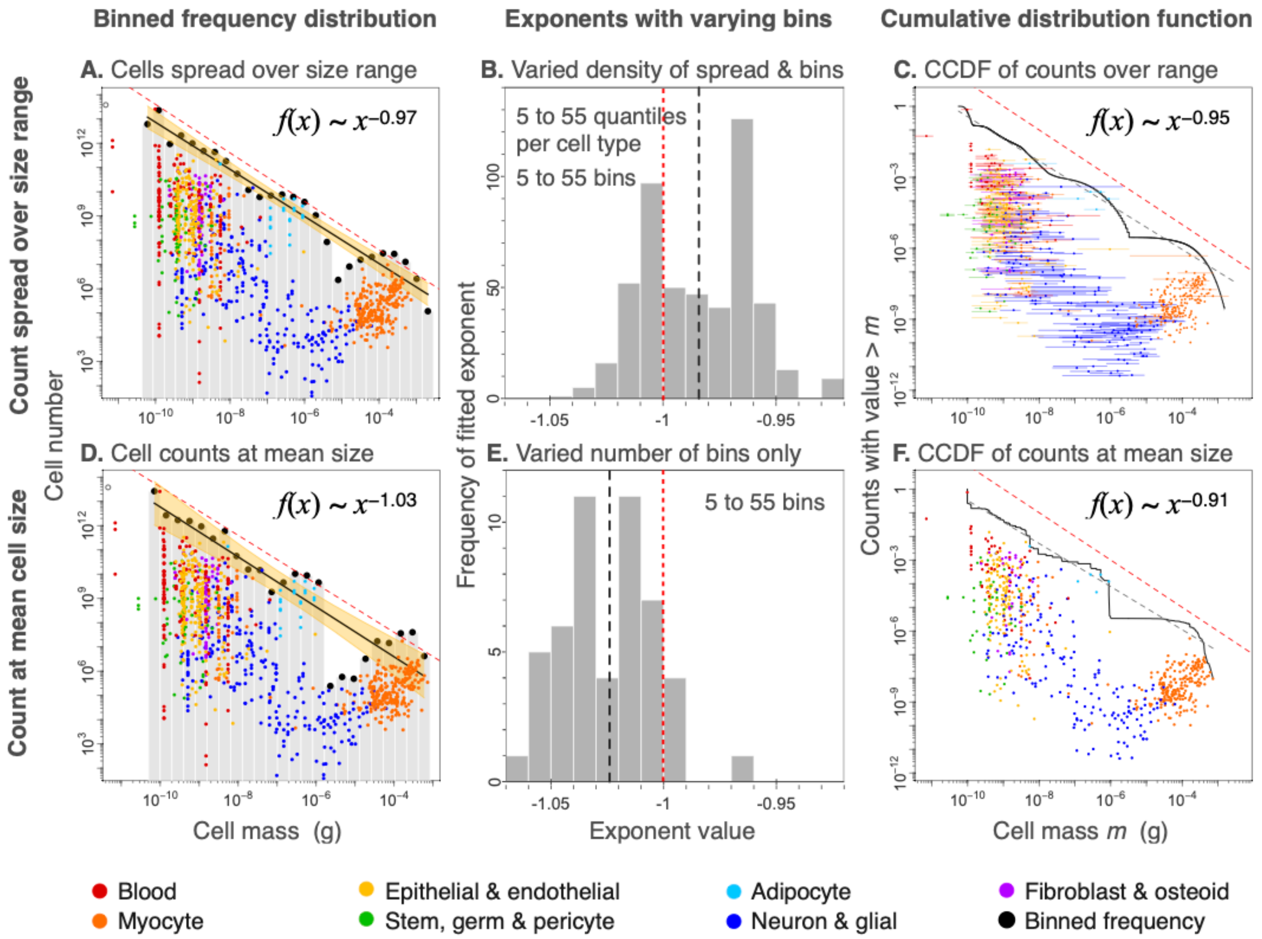

Fig. S4. Alternative characterizations of the cell size distribution. Top panels **A** to **C** show the cell size distribution with lognormally distributed counts for each cell group. Bottom panels **D** to **F** show only the counts associated with the mean cell size, without distributing the counts out from the mean. Panel **A** shows the same plot as Fig. 3, but all 1264 cell groups (colored points) are shown. Panel **D** shows the fit obtained from using the counts associated with the mean cell group size (without spreading counts across cell type size ranges). The fit is less regular (broader 95% confidence band) and slightly more negative exponent value). The precise exponent value varies depending on the number of quantiles for each cell type, and on the number of bins into which these quantiles are distributed. Panel **B** shows the exponents obtained from varying the size classes within and across cell types (both variables range from 5 to 55). Panel **E** varies only the number of bins across cell types, giving steeper exponent values (black dashed lines in B and E are mean exponents). Plots in A and D use octave size classes (changing by a factor of 2) giving 26 bins, with exponents near the respective mean exponent values in B and E. The histograms in B and E show there is no systematic relation between the number of bins and the exponent value. Finally, we can estimate the complementary cumulative distribution function (CCDF), equivalent to a rank-size plot (*sensu* Zipf's law). The colored points are for the same 700 cell groups shown in Fig. 3, rescaled by the body's ≈36 trillion cells. A true CCDF is not possible since the data come as counts associated with mean sizes (excluding cells smaller than erythrocytes) and we do not know the true sizes of all ≈36 trillion cells. Panel **C** distributes the counts lognormally over the size range of each cell group (shown by horizontal colored lines), as in A and Fig. 3. Both the least squares and maximum likelihood (following methods of Hanel et al. (40)) fit to the CCDF give exponents of –0.95. Panel **F** shows the CCDF for the counts at the mean size of the cell group (as in D). The red dashed lines (A to F) show a reference exponent of –1.

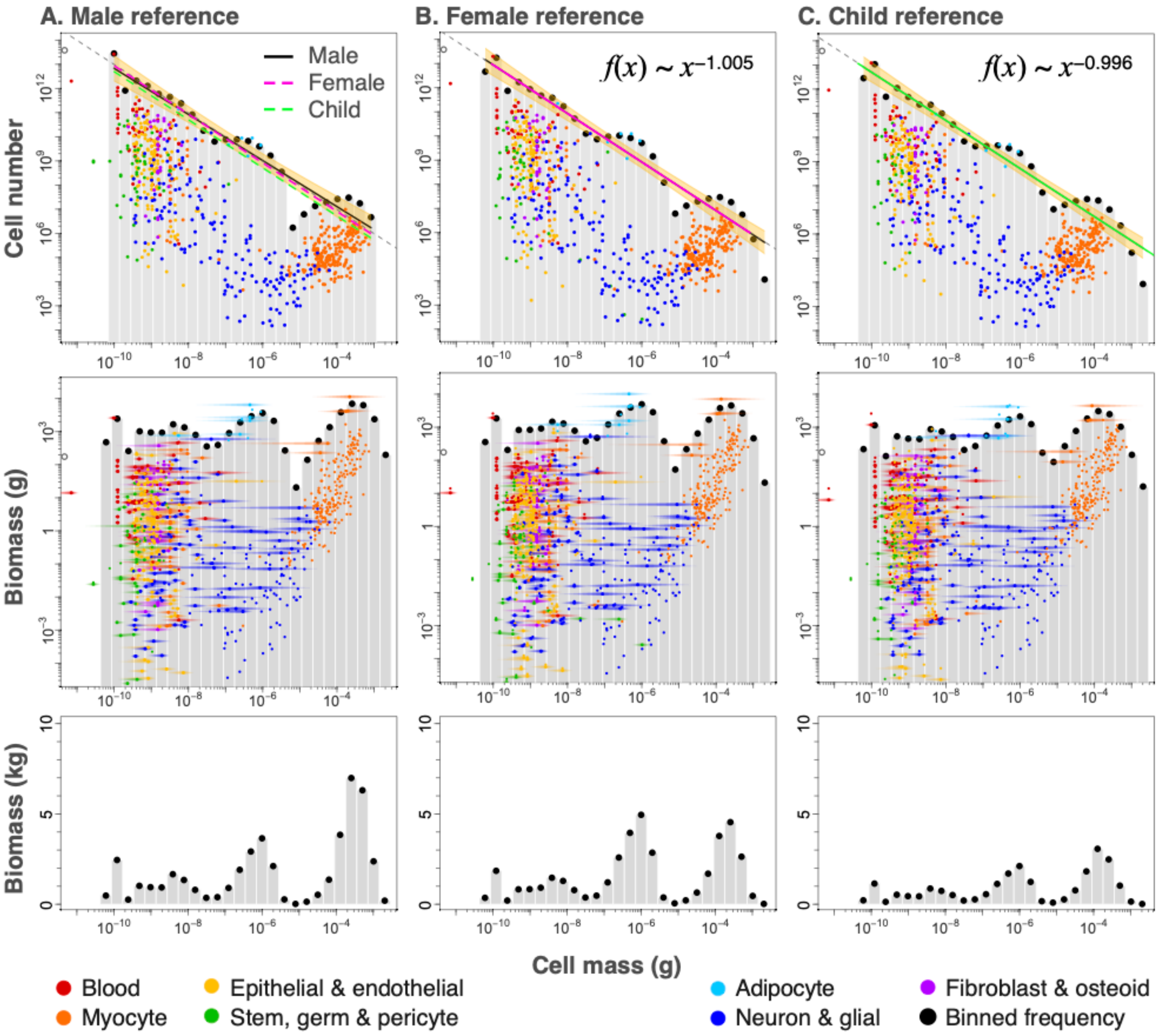

Fig. S5. Cell size-distributions across male, female and child models. Plots **A** to **C** show similar cell size-distributions with slopes very near –1. **A**. The data for the reference male are the same as shown in Fig. 3, and reproduced here for comparison, including the least squares best fit lines for reference female and child data. The gap between adipocytes and striated myocytes in the reference male is diminished in the reference female and shrinks further in the reference child of 10 years, as myocyte size is smaller in children. It is possible that this gap is further diminished in infants. The reference female has a larger peak in adipocytes 10^{-7} to 10^{-5} g due largely to breast tissue.

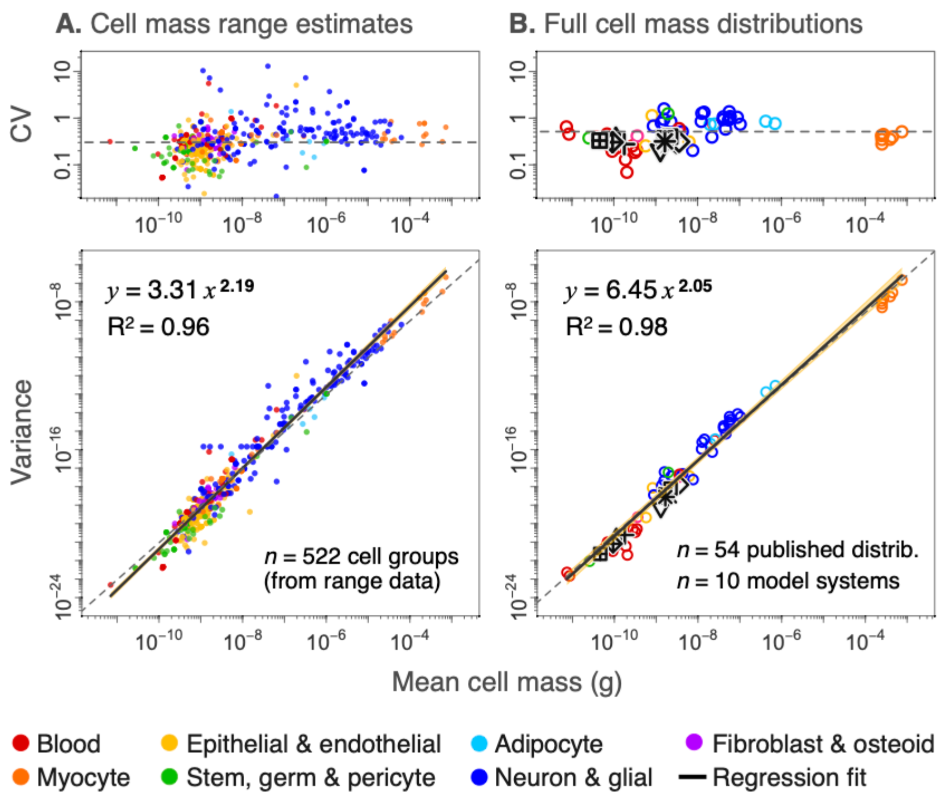

Fig. S6. Cell size variation across the human cell size range. The upper panels show the coefficient of variation (CV; standard deviation divided by the mean cell size) plotted vs. mean cell mass, with the dashed grey line showing the best fit with exponent of zero. The lower panels show cell size variance (standard deviation squared) plotted vs. mean cell mass, with the dashed grey line showing the best fit with an exponent of 2. Mean-variance scaling with exponent 2 is equivalent to a mass-invariant CV. **A**. Data derive from estimated range in cell mass derived from the literature for 522 cell groups. We assumed size range approximates four standard deviations. **B**. Data derive from 54 published size distributions, 30 of which are shown in Fig. 4 B and Fig. S2, and from 10 model systems in cell size research.

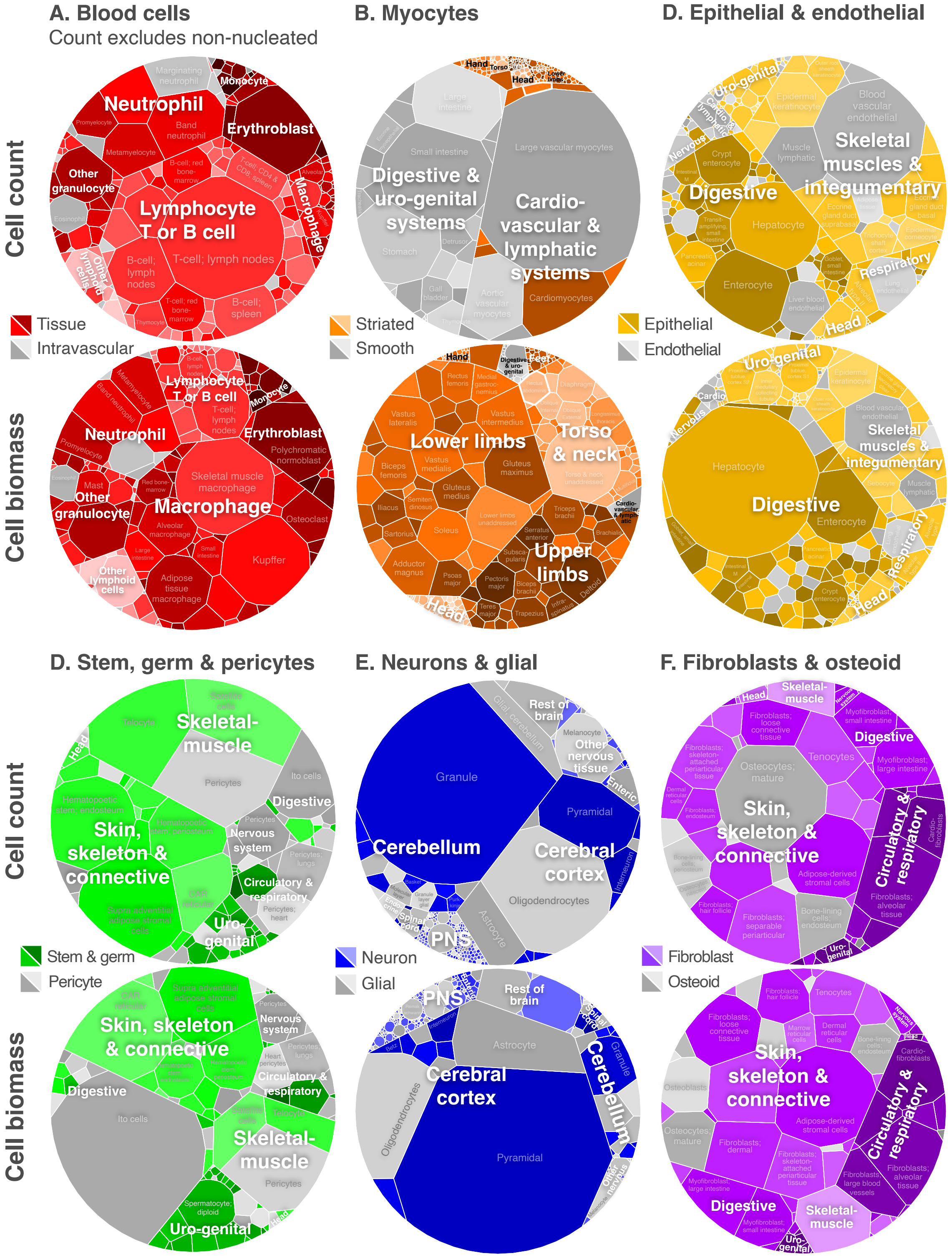

Fig. S7. Treemaps by major cell group. Voronoi treemaps for cell groups with count above, and biomass below. Blood cells are shown as tissue resident or circulating in the intravascular, and exclude non-nucleated erythrocytes and platelets (**A**). The remaining tree maps (**B** to **F**) are for other cell classes, showing cell types in different parts of the body. Further details at https://humancelltreemap.mis.mpg.de/

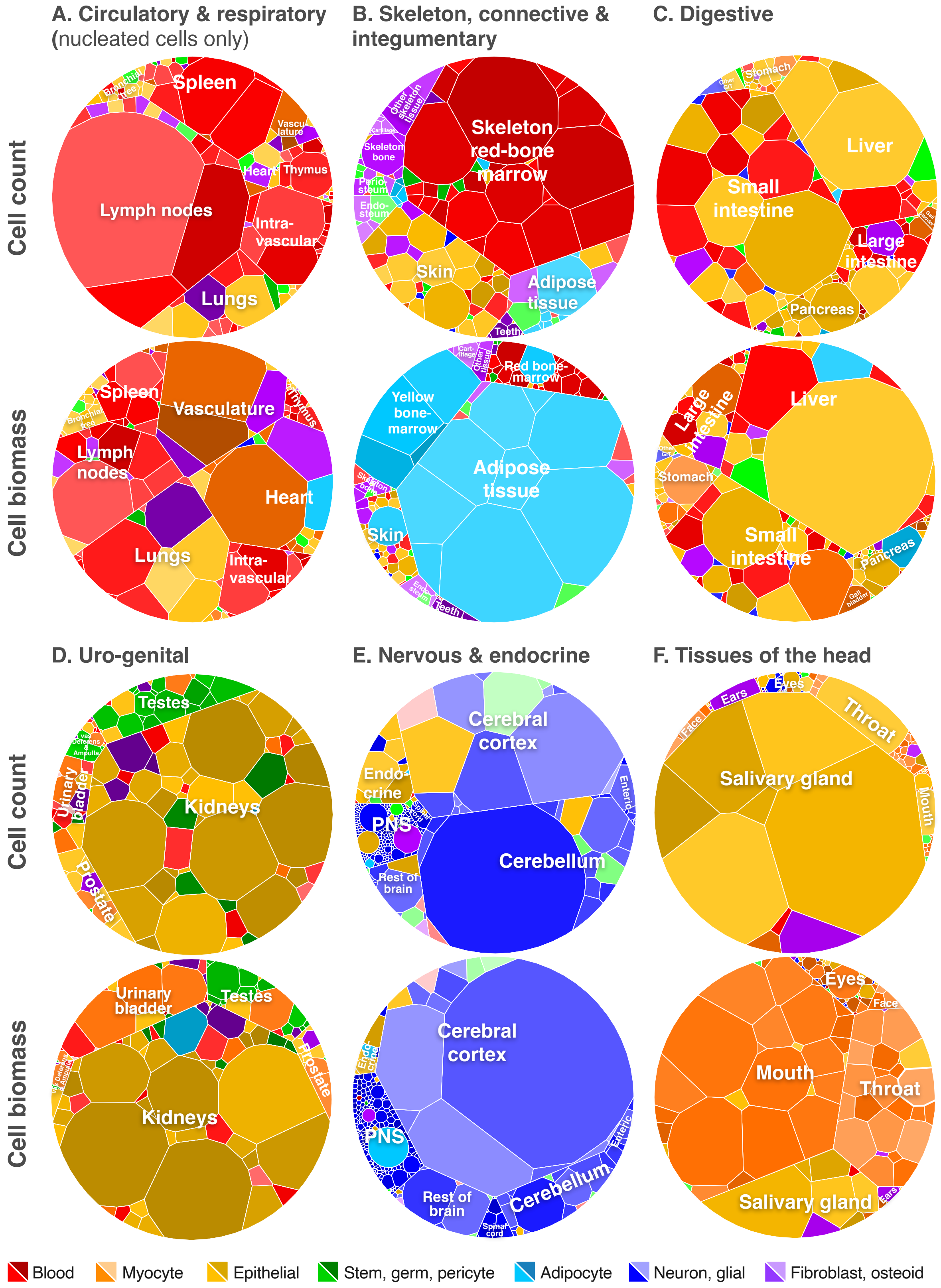

Fig. S8. Treemaps by organ systems. A to **F.** Data are same as Fig. S7, but grouped by organ and tissue systems. Treemaps can be explored at https://humancelltreemap.mis.mpg.de/

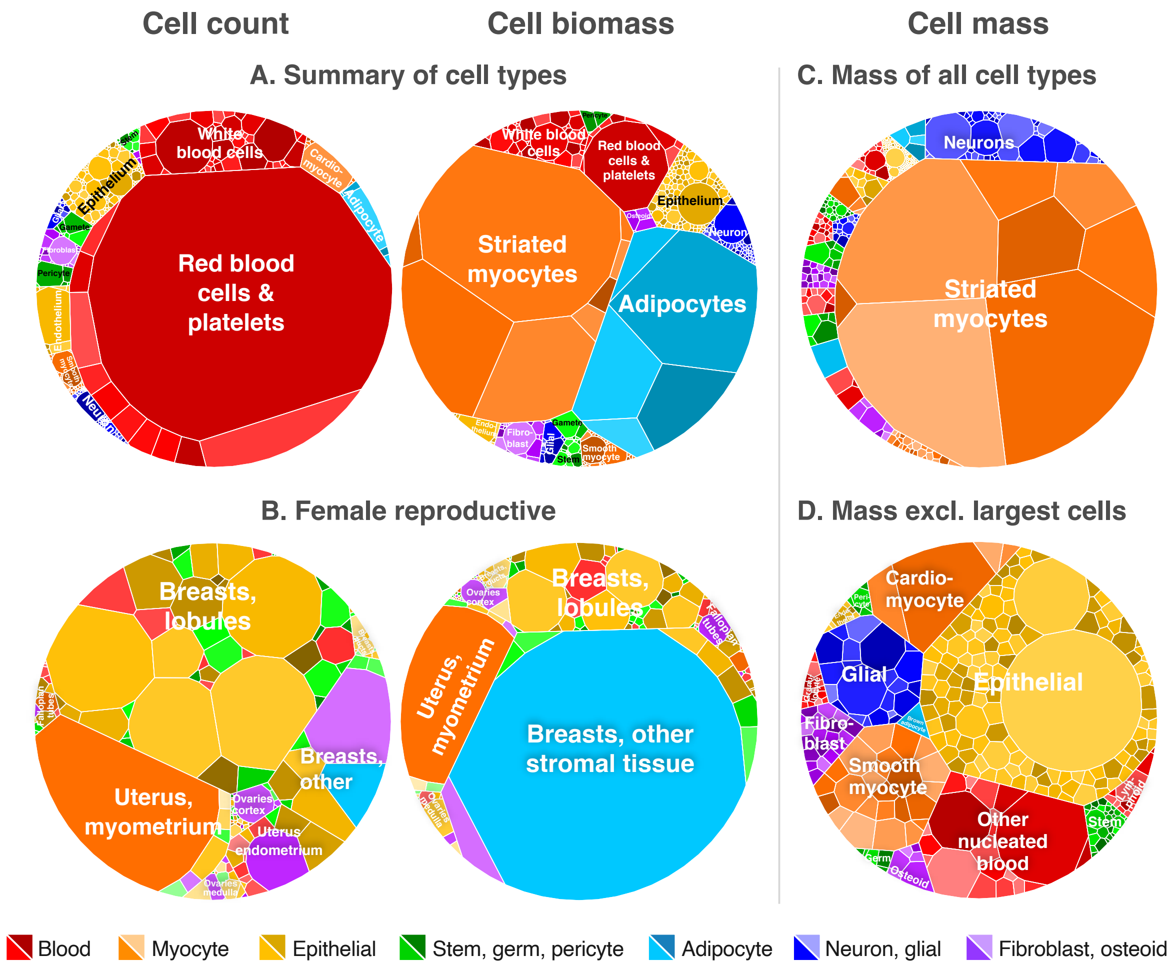

Fig. S9. Additional tree maps of interest. A. Cell count and biomass across distinct cell types, showing the same data categories as Fig. 1, but including all non-nucleated cells (left) and skeletal myocytes (center). **B**. Cell count and biomass across female reproductive organs and tissues, which are not included in any other tree maps since they represent the cells of a reference male (Fig. 1, and Figs. S7 and S8). **C.** Cell mass tree map showing the relative sizes of different cells (labels for different groups are shown in D). Skeletal myocytes dominate the sizes of all other cells, followed by peripheral nervous system neurons, and white adipocytes. **D.** Relative cell mass is shown with these largest cells excluded (skeletal myocytes, peripheral nervous system neurons and white adipocytes are excluded).

REFERENCES

1. J. P. Greer, *et al.*, *Wintrobe's clinical hematology*, 11th Ed. (Lippincott Williams & Wilkins, 2004).
2. K. Kaushansky, M. A. Lichtman, E. Beutler, *Williams hematology*, 8th Ed. (McGraw-Hill Medical, 2010).
3. G. P. Downey, *et al.*, Retention of leukocytes in capillaries: role of cell size and deformability. *Journal of applied physiology* **69**, 1767–1778 (1990).
4. M. Di Mascio, *et al.*, Noninvasive in vivo imaging of CD4 cells in simian-human immunodeficiency virus (SHIV)–infected nonhuman primates. *Blood* **114**, 328–337 (2009).
5. W. S. Snyder, *et al.*, *ICRP Publication 23: Report of the Task Group on Reference Man: A Report Prepared by a Task Group of Committee 2 of the International Commission on Radiological Protection* (Pergamon Oxford, 1975).
6. J. Valentin, *ICRP Publication 89: Basic anatomical and physiological data for use in radiological protection: reference values* (Elsevier Health Sciences, 2003).
7. E. Bianconi, *et al.*, An estimation of the number of cells in the human body. *Annals of human biology* **40**, 463–471 (2013).
8. R. Sender, S. Fuchs, R. Milo, Revised estimates for the number of human and bacteria cells in the body. *PLoS biology* **14**, e1002533 (2016).
9. F. Trepel, Number and distribution of lymphocytes in man. A critical analysis. *Klin Wochenschr* **52**, 511–515 (1974).
10. A. C. Paul, N. Rosenthal, Different modes of hypertrophy in skeletal muscle fibers. *Journal of Cell Biology* **156**, 751–760 (2002).
11. H. W. Vliegen, A. Van der Laarse, C. J. Cornelisse, F. Eulderink, Myocardial changes in pressure overload-induced left ventricular hypertrophy: A study on tissue composition, polyploidization and multinucleation. *European heart journal* **12**, 488–494 (1991).
12. C.-P. Adler, H. Friedburg, G. W. Herget, M. Neuburger, H. Schwalb, Variability of cardiomyocyte DNA content, ploidy level and nuclear number in mammalian hearts. *Virchows Archiv* **429**, 159–164 (1996).
13. M. Mollova, *et al.*, Cardiomyocyte proliferation contributes to heart growth in young humans. *Proceedings of the National Academy of Sciences* **110**, 1446–1451 (2013).
14. Y. Guo, W. T. Pu, Cardiomyocyte maturation: new phase in development. *Circulation research* **126**, 1086–1106 (2020).
15. O. Bergmann, *et al.*, Dynamics of cell generation and turnover in the human heart. *Cell* **161**, 1566–1575 (2015).
16. H.-G. Menzel, C. Clement, P. DeLuca, ICRP Publication 110. Realistic reference phantoms: an ICRP/ICRU joint effort. A report of adult reference computational phantoms. *Ann ICRP* **39**, 1–164 (2009).
17. D. W. Fawcett, W. Bloom, E. Raviola, J. B. Angevine, A. Maximov, *Bloom and Fawcett, A textbook of histology.* (Chapman & Hall, 1994).
18. M. H. Ross, W. Pawlina, *Histology* (Lippincott Williams & Wilkins, 2006).

19. R. A. Freitas, *Nanomedicine, volume I: basic capabilities* (Landes Bioscience Georgetown, TX, 1999).
20. S. Laforest, *et al.*, Comparative analysis of three human adipocyte size measurement methods and their relevance for cardiometabolic risk. *Obesity* **25**, 122–131 (2017).
21. B. P. Leitner, *et al.*, Mapping of human brown adipose tissue in lean and obese young men. *Proceedings of the national academy of sciences* **114**, 8649–8654 (2017).
22. H. Sacks, M. E. Symonds, Anatomical locations of human brown adipose tissue: functional relevance and implications in obesity and type 2 diabetes. *Diabetes* **62**, 1783–1790 (2013).
23. F. A. C. Azevedo, *et al.*, Equal numbers of neuronal and nonneuronal cells make the human brain an isometrically scaled-up primate brain. *Journal of Comparative Neurology* **513**, 532–541 (2009).
24. H. Duvernoy, S. Delon, J. L. Vannson, The vascularization of the human cerebellar cortex. *Brain research bulletin* **11**, 419–480 (1983).
25. L. Xuezheng, *et al.*, Quantitative analysis of density of capillaries in human cerebellum. *Chinese Journal of Anatomy* **20**, 253–256 (1997).
26. F. Lauwers, F. Cassot, V. Lauwers-Cances, P. Puwanarajah, H. Duvernoy, Morphometry of the human cerebral cortex microcirculation: general characteristics and space-related profiles. *Neuroimage* **39**, 936–948 (2008).
27. C. N. Hall, *et al.*, Capillary pericytes regulate cerebral blood flow in health and disease. *Nature* **508**, 55–60 (2014).
28. I. R. Murray, *et al.*, Skeletal and cardiac muscle pericytes: Functions and therapeutic potential. *Pharmacology & therapeutics* **171**, 65–74 (2017).
29. S. J. Mills, A. J. Cowin, P. Kaur, Pericytes, mesenchymal stem cells and the wound healing process. *Cells* **2**, 621–634 (2013).
30. H. H. Mitchell, T. S. Hamilton, F. R. Steggerda, H. W. Bean, The chemical composition of the adult human body and its bearing on the biochemistry of growth. *Journal of Biological Chemistry* **158**, 625–637 (1945).
31. R. Mlcoo. Forbes, A. R. Cooper, H. H. Mitchell, The composition of the adult human body as determined by chemical analysis. *Journal of Biological Chemistry* **203**, 359–366 (1953).
32. R. M. Forbes, H. H. Mitchell, A. R. Cooper, Further studies on the gross composition and mineral elements of the adult human body. *Journal of Biological Chemistry* **223**, 969–975 (1956).
33. J. M. Barkmeier, E. S. Luschei, Quantitative analysis of the anatomy of the epineurium of the canine recurrent laryngeal nerve. *The Journal of Anatomy* **196**, 85–101 (2000).
34. J. Bahney, C. S. von Bartheld, The cellular composition and glia–neuron ratio in the spinal cord of a human and a nonhuman primate: comparison with other species and brain regions. *The Anatomical Record* **301**, 697–710 (2018).
35. B. Alberts, *et al.*, *Molecular Biology of the Cell*, 3rd Ed. (Garland Science, 1994).
36. M. K. Vickaryous, B. K. Hall, Human cell type diversity, evolution, development, and classification with special reference to cells derived from the neural crest. *Biol Rev Camb Philos Soc* **81**, 425–455 (2006).

37. D. Osumi-Sutherland, *et al.*, Cell type ontologies of the Human Cell Atlas. *Nat Cell Biol* **23**, 1129–1135 (2021).
38. S. Mohan, I. Coto Hernández, M. K. Selig, S. Shibata, N. Jowett, Stain-free resolution of unmyelinated axons in transgenic mice using fluorescence microscopy. *Journal of Neuropathology & Experimental Neurology* **78**, 1178–1180 (2019).
39. I. B. ICRP Publication 70, *Physiological Data for use in Radiological Protection-The Skeleton* (Oxford; Published for the International Commission on Radiological Protection by Elsevier Health Sciences; transferred to SAGE Publications Ltd, 1995).
40. R. Hanel, B. Corominas-Murtra, B. Liu, S. Thurner, Fitting power-laws in empirical data with estimators that work for all exponents. *PloS one* **12**, e0170920 (2017).
